# Targeted delivery platforms for *in vivo* immune cell engineering: emerging strategies for cancer immunotherapy

**DOI:** 10.3389/fphar.2026.1936688

**Published:** 2026-09-21

**Authors:** Hari Krishnareddy Rachamala, Sandeep Atla, Ramcharan Singh Angom

**Affiliations:** 1 Department of Biochemistry and Molecular Biology, Mayo Clinic College of Medicine and Sciences, Jacksonville, FL, United States; 2 Texas A&M Drug Discovery Center and Department of Chemistry, College of Arts and Sciences, Texas A&M University, College Station, TX, United States

**Keywords:** cancer immunotherapy, chimeric antigen receptor (CAR) therapy, *In vivo* immune cell engineering, lipid nanoparticles, mRNA therapeutics, targeted delivery

## Abstract

Cancer immunotherapy is a transformative technology allowing for the elimination of malignant cells through immune system stimulation; however, the large-scale clinical application of adoptive cellular therapy technologies is limited due to difficulties in *ex vivo* immune cell engineering, high costs, and complicated manufacturing process. Recently, *in vivo* immune cell engineering was discovered as an emerging approach that allows for the genetic reprogramming of immune cells in patients, thus circumventing many problems inherent to traditional cellular therapy technologies. Breakthroughs in targeted drug delivery systems such as cell-penetrating peptides, antibody- and receptor-mediated delivery approaches, lipid nanoparticles (LNPs), polymeric drug carriers, and biomimetic delivery systems allow for the more effective selective delivery of messenger RNA (mRNA), clustered regularly interspaced short palindromic repeats (CRISPR)/Cas gene editing systems, proteins, and other types of cargo to the target immune cells. *In vivo* immune cell engineering allows for the efficient generation of chimeric antigen receptor T-cells (CAR-T), CAR- natural killer (NK) cells, engineered macrophages, dendritic cells, and other immune cell types; moreover, transient and permanent immune reprogramming is achievable via vivo technologies. The present review discusses the biological barriers limiting immune cell-targeted delivery, including biodistribution, cellular uptake, intracellular transport, and endosomal escape of delivered drugs, and reviews the latest advancements in targeted delivery technologies addressing these obstacles. We also address the latest applications of *in vivo* immune cell engineering in hematological malignancies and solid tumors, including mRNA therapeutics, CRISPR-based genome editing, and advanced programmable immunotherapy technologies. Furthermore, we discuss unresolved issues related to the specificity of targeting, efficiency of drug delivery, safety of treatment, manufacturing processes, and regulation of *in vivo* immune engineering technologies and outline future opportunities arising from the integration of nanomedicine, synthetic biology, genome engineering, and artificial intelligence-assisted materials science.

## Graphical Abstract

**Figure:**
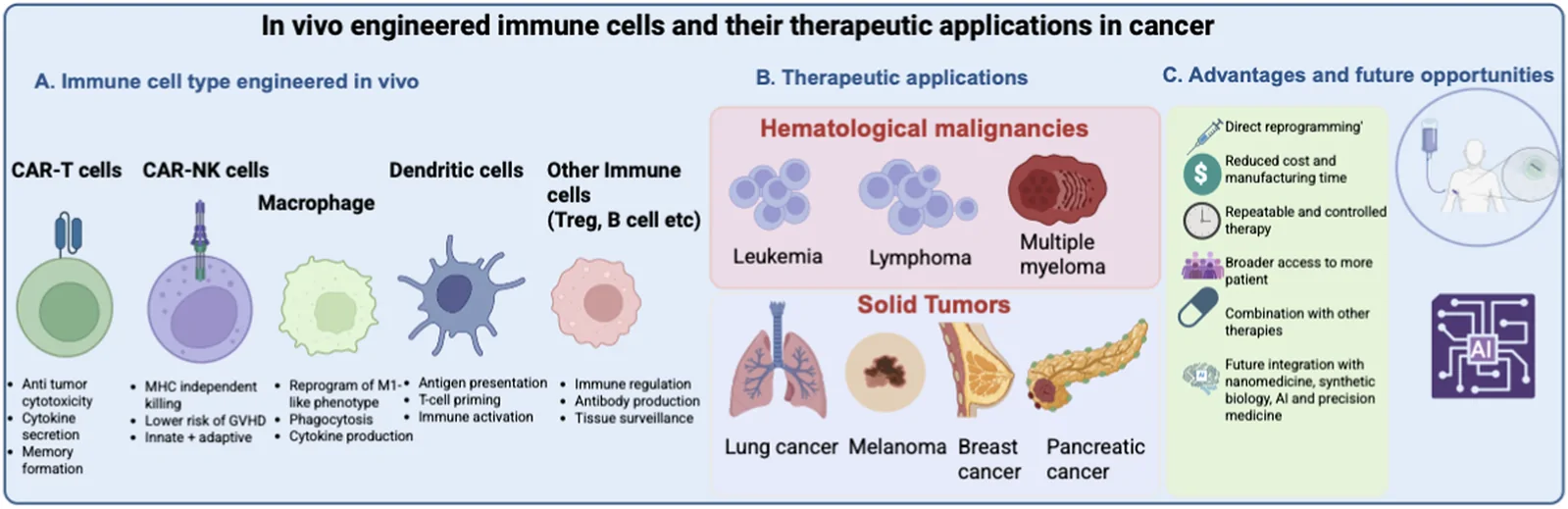
*In vivo* engineered immune cells and their therapeutic applications in cancer. Schematic overview of current strategies and therapeutic opportunities for direct *in vivo* immune-cell engineering. **(A)** Major immune-cell populations that can be engineered *in vivo* include CAR-T cells, CAR-NK cells, macrophages, dendritic cells, and other immune cells such as regulatory T (Treg) and B cells. Their major therapeutic functions include tumor-specific cytotoxicity, cytokine secretion, immune memory, MHC-independent killing, macrophage repolarization, enhanced phagocytosis, antigen presentation, T-cell priming, and modulation of immune responses. **(B)** *In vivo* engineered immune cells have potential applications in both hematological malignancies, including leukemia, lymphoma, and multiple myeloma, and solid tumors, including lung cancer, melanoma, breast cancer, and pancreatic cancer. **(C)** Key advantages and future opportunities include direct immune-cell reprogramming within the patient, reduced manufacturing time and cost, repeatable and controllable dosing, broader patient accessibility, compatibility with combination therapies, and integration with nanomedicine, synthetic biology, artificial intelligence, and precision medicine. Created with BioRender.

## 1 Introduction

Immunotherapy for cancer is a revolution in treating cancer because it makes use of the body’s natural ability to detect and destroy cancerous cells. Unlike other traditional methods of treatment such as chemotherapy and radiation therapy that rely on destroying cancer cells through their high proliferation rate, immunotherapy enhances the natural ability of the body to destroy cancer cells (Zhang M. et al., 2025). In the last 10 years, immune checkpoint inhibition, adoptive cell transfer, therapeutic cancer vaccines, and immune stimulation have resulted in remarkable clinical successes for a variety of tumors, making immunotherapy a cornerstone of cancer therapy (Chen et al., 2025a). In all these strategies, genetic engineering of immune cells, especially CAR-T cells, has shown great effectiveness in treating hematologic cancers and has established genetic engineering of immune cells as an effective therapeutic approach (Jiang et al., 2025). Although there have been several advances in this field, widespread adoption of adoptive cellular therapy faces limitations due to their reliance on the *ex vivo* process of production. Current CAR-T and other adoptive cellular therapies rely on harvesting autologous or allogeneic immune cells, genetically modifying them under GMP conditions, expanding them *ex vivo*, and performing rigorous quality control prior to reinfusion. This limits the application of these therapies due to logistical and financial constraints on access (Dan and Kang-Zheng, 2025). Further complexities related to the effectiveness of transplanted immune cells arise in the treatment of solid tumors where there are many biological obstacles to effective therapy. These include deposition of dense extracellular matrix, tumor blood vessels that do not develop normally, heterogenous antigen expression, tumor hypoxia, and an immunosuppressive environment within the tumor itself (Zhang J. et al., 2025). Moreover, repeated antigen stimulation in the tumor setting leads to dysfunction and cell exhaustion of the immune cells. Overcoming these obstacles has thus become one of the goals of designing future immunotherapy (Wu et al., 2026).

New developments in the fields of nanomedicine, biomaterial science, and nucleic acid therapy have resulted in in vivo immune cell engineering being developed as a new treatment approach, which involves direct genetic reprogramming of immune cells in a living body (Liu Y. et al., 2026). By directly delivering therapeutic cargos, including small interfering RNA (siRNA), CRISPR/Cas gene-editing systems, and other nucleic acid therapeutics, this approach has the potential to simplify treatment, reduce costs, accelerate therapy, and expand access to a much larger patient population (Naqvi et al., 2026). Moreover, in addition to making immune cell therapies more scalable, *in vivo* engineering will make possible to administer immune cell treatment repeatedly and regulate the functionality of the cells (Yang et al., 2026). The advances made in this rapidly progressing field can be attributed to advancements in delivery technologies. The development of LNPs stands out as the main non-viral technology for nucleic acid delivery after their proven efficiency in mRNA delivery and the development of technologies for delivering these particles specifically into immune cell populations (Wu et al., 2024). Advances in the development of CCPs and antibodies or receptors for targeting purposes, along with advances in polymeric nanoparticles and biomimetics, have added new possibilities in the design of delivery technologies that will deliver therapeutic cargo to T cells, NK cells, dendritic cells, macrophages, and other immune cell populations (Prakash et al., 2025). Nonetheless, the efficient engineering of immune cells *in vivo* is still a daunting task. After systemic delivery, the delivery system needs to cross many biological barriers such as nonspecific biodistribution, fast elimination by the mononuclear phagocytic system, poor tissue penetration, inefficient cellular internalization, entrapment in endosomes, and incomplete cargo release in the cytosol (Hegde et al., 2025; Siebart et al., 2024). Furthermore, it is necessary to deliver efficiently to the immune cells while avoiding unwanted delivery to other tissues, being nonimmunogenic, noninflammatory, and safe in the long term.

In this review, we provide an overview of the new generation of targeted delivery platforms that have been fueling the development of vivo immune cell engineering for cancer immunotherapy. In the first place, we describe the key biological obstacles for immune-cell-targeted delivery and then review the state-of-the-art in cell-penetrating peptides, antibody- and receptor-targeting delivery systems, LNPs, polymeric nanoparticles, and biomimetic delivery carriers. In addition, we cover the application of the reviewed techniques for the development of immune cells with improved functional properties, such as CAR-T cells, CAR-NK cells, and CRISPR- and mRNA-based immune cells reprogramming approaches. Finally, we analyze the status of the development of clinically translatable delivery approaches, open challenges, and perspectives for the integration of delivery technology and synthetic biology approaches into precision immunotherapy. An overview comparing conventional *ex vivo* immune cell engineering with emerging *in vivo* immune cell engineering approaches is illustrated in Figure 1.

**FIGURE 1:**
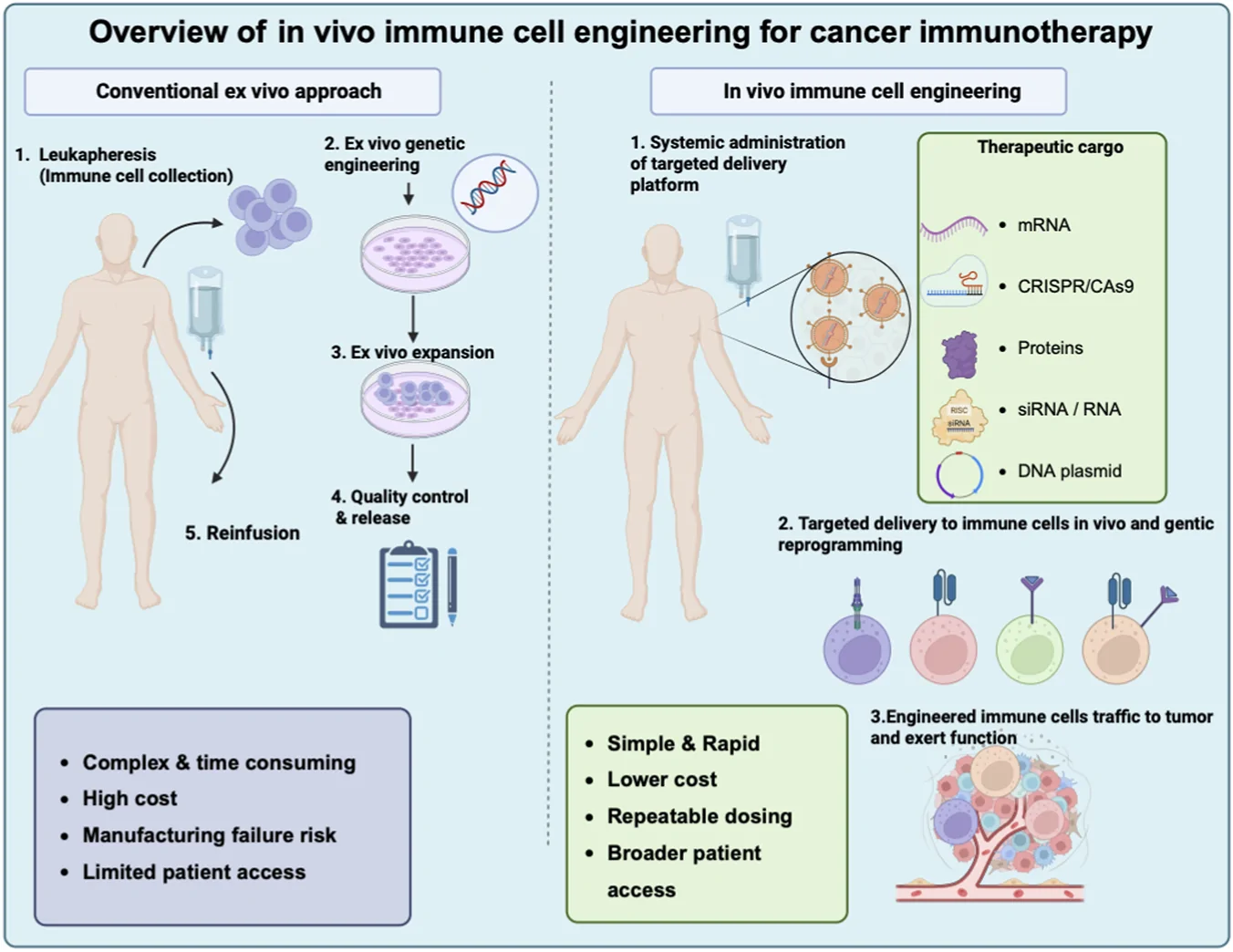
Comparison of *ex vivo* and *in vivo* immune cell engineering approaches for cancer immunotherapy. The conventional *ex vivo* approach involves multiple sequential steps, including leukapheresis for immune-cell collection, genetic modification of isolated cells, *ex vivo* expansion, quality-control testing and product release, followed by reinfusion into the patient. These procedures are complex, time-consuming, costly, and may limit patient accessibility. In contrast, *in vivo* immune-cell engineering uses targeted delivery platforms administered systemically to deliver therapeutic cargo directly to endogenous immune cells. Cargoes may include mRNA, CRISPR/Cas9 components, proteins, siRNA/RNA, or DNA plasmids, enabling transient or durable genetic and functional reprogramming. Following engineering, modified immune cells can traffic to tumors and exert antitumor activity. This approach may simplify manufacturing, reduce treatment time and cost, enable repeatable dosing, and broaden patient access to engineered immune-cell therapies. Created with BioRender.

## 2 Biological barriers to immune cell-targeted delivery

The effectiveness of *in vivo* immune cell targeting through engineering relies greatly on the capacity of the delivery vehicles to navigate biological barriers that hinder effective delivery of the cargo to their target immune cells (Lei et al., 2026). Once the systemically administered nanocarriers evade the mononuclear phagocyte system, it is important for them to be able to distribute themselves appropriately, recognize the target immune cells, be internalized effectively, escape endosomes and deliver the cargo to the correct intracellular destination (Casimiro et al., 2026). The inability to navigate biological barriers may greatly affect delivery efficiency and even safety. Recent advances in immune cell-targeted delivery have demonstrated that rational engineering of LNPs can substantially improve targeting specificity and intracellular delivery. For example, antibody-capture strategies and optimized lipid nanoparticle formulations have enabled highly selective delivery of mRNA and CRISPR cargo to T cells, thereby enhancing *in vivo* immune cell engineering while minimizing off-target effects (Chen M. Z. et al., 2025; Lu et al., 2025). After uptake by cells, efficient transport and escape from endosomes become essential for delivering nucleic acids. Cargo entrapment inside the endosome is often associated with activation of inflammatory pathways due to lack of access of the cargo to the cytosol. Recent studies have also demonstrated that rational lipid design can improve endosomal escape while minimizing inflammatory responses. These findings emphasize the importance of engineering intracellular trafficking pathways to enhance cytosolic delivery of therapeutic nucleic acids (Omo-Lamai et al., 2025). Similarly, high-throughput screening and machine learning-guided nanoparticle optimization have enabled the identification of spleen-targeting LNPs capable of efficiently delivering genome-editing cargos to immune cells *in vivo*, highlighting the potential of computationally guided nanomedicine design (Lian et al., 2025).

In addition to the delivery of therapeutic nucleic acids, recent advancements in gene editing technologies have made progress in the application of targeted delivery techniques for *in vivo* genome editing of the immune cells. In one study, Nyberg et al. designed a dual-vector system where the EDVs targeted against CD3 deliver Cas9 ribonucleoprotein complex, and an AAV-hT7 vector, targeting the T cells, delivered the CAR donor DNA, resulting in site-specific integration at TRAC locus. *In vitro* results showed ∼39.2% CAR^+^TCR^−^ T cells in the EDV/AAV approach while TRAC-CAR-T cells were found in 3% of splenic T cells. With the help of the optimized anti-CD3 targeted EDVs along with the evolved AAV-hT7 capsid, engineered CAR-T cells showed expansion and showed B-cell aplasia and antitumor activities in various cancer mouse models such as B-cell leukemia, multiple myeloma, and solid tumors. This system requires both the delivery vectors to be successfully delivered into the same T cell, and integration in homologous manner favors only proliferating cells. Moreover, the presence of neutralizing antibodies can influence AAV-mediated gene transfer, and this system increases the complexities of vector manufacturing and delivery (Nyberg et al., 2026).

Together, these research findings indicate that effective engineering of immune cells *in vivo* relies on the simultaneous optimization of all steps of the delivery process from the immune cell targeting to the endosomal escape and subsequent genome editing. The future progress in biomaterials science, chemistry of nanoparticles, and computational guidance of delivery vehicles development should contribute to improvement of these aspects, making the immune cells delivery more efficient and safer for patients.

### 2.1 Biodistribution and targeting specificity

After being administered systematically, the carriers face many physiological barriers that greatly affect their ability to get to and effectively manipulate target immune cells. Rapid uptake by the liver, spleen, and mononuclear phagocyte system (MPS), along with renal excretion, adsorption by serum proteins, and non-specific distribution into different tissues, often results in decreasing the amount of cargo that can be used to target immune cells (Zelepukin et al., 2024). Moreover, the immune cells have very active migration dynamics and constantly move between blood, secondary lymphoid organs, and peripheral tissues; therefore, it is difficult to deliver cargo only to certain types of immune cells. This is one of the main problems in immune cell engineering (Jan et al., 2026). Immune cell surface target choice is another important factor that determines the selectivity of the delivery system. Concerning targeting T cells, common targets include CD3, CD4, CD5, CD7, and CD8, whereas for dendritic cells and macrophages, lineage-specific targets or ligands, e.g., involved in mannose and phosphatidylserine recognition pathways, can be used for targeting. Apart from targeting through receptors, organ-based biodistribution with selective enrichment in lymphoid organs such as spleen can provide additional access to the immune cell subsets (Tables 1, 2).

**TABLE 1:** Overview of targeted delivery platforms for *in vivo* immune cell engineering.

Delivery platform	Major cargo	Target immune cells	Key advantages	Major limitations	References
CPPs	CRISPR RNPs, mRNA, proteins	T cells, NK cells, HSPCs	Non-viral delivery, efficient intracellular transport	Limited targeting specificity, endosomal trappin	(Foss et al., 2023; Zhang et al., 2024)
Antibody/Receptor-mediated targeting	mRNA, DNA, proteins	T cells, NK cells, DCs	High cell specificity, receptor-mediated uptake	Receptor heterogeneity, antibody complexity	(Chen J. et al., 2025; Lutz et al., 2023; Hagelstein et al., 2021; Zekri et al., 2023)
Lipid nanoparticles (LNPs)	mRNA, siRNA, DNA, CRISPR	T cells, NK cells, macrophages, DCs	Clinically advanced, scalable, high transfection	Liver accumulation, PEG immunogenicity	(Billingsley et al., 2024; Li T. et al., 2024; Zhou et al., 2022; Bimbo et al., 2025; Zhao et al., 2025b)
Polymeric nanoparticles	mRNA, siRNA, proteins	DCs, macrophages, T cells	Tunable properties, controlled release	More complex formulation	(Yuan et al., 2024; Zhang Y. et al., 2025; Ben-Akiva et al., 2023; Wang et al., 2025)
Biomimetic delivery systems	mRNA, proteins, STING agonists	T cells, macrophages, DCs	Excellent biocompatibility and immune targeting	Difficult large-scale manufacturing	(Chen et al., 2025b; Zhao et al., 2025b; Du et al., 2026; Huo et al., 2026; Zhang et al., 2026b)
Viral and virus-derived vectors	DNA/transgenes, CRISPR–Cas9 RNPs, gene-editing components	T cells, NK cells	High transduction efficiency, durable gene expression, engineered cell-specific targeting	Antiviral immunity, cargo limitations, repeat-dosing constraints, manufacturing complexity, insertional/genomic safety concerns	(Andorko et al., 2025; Hamilton et al., 2024; Nyberg et al., 2026)

Comparison of major delivery platforms, representative therapeutic cargoes, target immune cell populations, key advantages, major limitations, and selected references. Abbreviations: AAV, adeno-associated virus; CAR, chimeric antigen receptor; CPPs, cell-penetrating peptides; CRISPR, clustered regularly interspaced short palindromic repeats; DCs, dendritic cells; EDVs, enveloped delivery vehicles; HSPCs, hematopoietic stem and progenitor cells; LNPs, lipid nanoparticles; NK, natural killer; PEG, polyethylene glycol; RNPs, ribonucleoproteins; siRNA, small interfering RNA; STING, stimulator of interferon genes.

**TABLE 2:** Targeted delivery systems for *in vivo* immune cell engineering.

Delivery system	Target cell	Immune-cell targeting ligand/Receptor	Therapeutic cargo	Major outcome	Cancer/Application	Reference
PERC peptide delivery	T cells, B cells, NK cells	No cell-specific targeting ligand; CPP-mediated intracellular delivery	CRISPR/Cas9 RNP	Efficient genome editing and CAR integration	Immune cell engineering	(Foss et al., 2023)
PAGE CPP platform	T cells, HSPCs	No cell-specific targeting ligand; CPP/endosomal-escape peptide-mediated delivery	Cas9/Cas12a RNP	Up to 98%–100% genome editing	CAR-T engineering	(Zhang et al., 2024)
Fc-binding antibody-capture LNP	T cells	CD3 (anti-CD3 antibody)	mRNA	Highly selective mRNA delivery	*In vivo* immune engineering	(Chen M. Z. et al., 2025)
CD3-targeted Ab-LNP	T cells	CD3 (anti-CD3 antibody)	CAR mRNA	Functional CAR-T generation	B-cell malignancies	(Billingsley et al., 2024)
NCtx LNP	T cells	CD3/CD7 dual targeting	CAR DNA + transposase mRNA	Stable CAR integration	Leukemia	(Bimbo et al., 2025)
CD3-targeted LNP + IL-7	T cells	CD3 (anti-CD3 antibody)	CAR mRNA + IL-7 mRNA	Improved persistence and antitumor activity	Melanoma	(Li H. et al., 2026)
Dendritic cell lipopolyplex	Dendritic cells	DC-directed lipopolyplex; no T/NK targeting	mRNA vaccine	Strong CD8^+^ T-cell activation	Cancer vaccination	(Van der Jeught et al., 2018)
DOTAP dual liposomes	NK cells	No receptor-specific targeting ligand reported; DOTAP-mediated NK-cell delivery	CAR mRNA	Enhanced CAR-NK cytotoxicity	Hematologic cancers	(Shin et al., 2024)
PSβ-LNP	Macrophages	Macrophage-directed formulation; no T/NK targeting	CAR mRNA	M1 polarization and CAR macrophages	Solid tumors	(Gu et al., 2025)
Fluorinated polymeric NP	Splenic immune cells	Spleen-tropic/protein-corona-mediated targeting; no specific T/NK receptor ligand	mRNA	Efficient splenic immune-cell transfection	Melanoma	(Zhang Y. et al., 2025)
Personalized hydrogel vaccine	Dendritic cells	No T/NK-specific targeting ligand	Tumor antigens + STING agonist	Durable T-cell immunity	Melanoma, Breast cancer	(Wang D. et al., 2026)
DC-derived exosomes	T cells	Indirect T-cell activation; no direct T-cell targeting ligand specified	IL-12 + anti-CTLA-4	Enhanced antitumor immunity	Melanoma, Lung cancer	(Chen J. et al., 2025)
Macrophage membrane-coated NP	TAMs	Macrophage membrane-mediated tumor/TAM interaction; no T/NK targeting	TMP195	M2-to-M1 repolarization	Breast cancer	(Zhao et al., 2025a)
Activated T-cell membrane liposome	Multiple immune cells	Activated T-cell membrane-mediated targeting; no single receptor specified	STING agonist	Enhanced innate and adaptive immunity	Solid tumors	(Du et al., 2026)

Representative targeted delivery systems for *in vivo* immune cell engineering, highlighting target immune cells, immune-cell targeting ligand/receptor, therapeutic cargo, major outcomes, cancer applications, and selected references. Abbreviations: CAR, chimeric antigen receptor; RNP, ribonucleoprotein; LNP, lipid nanoparticle; CPP, cell-penetrating peptide; HSPCs, hematopoietic stem and progenitor cells; TAMs, tumor-associated macrophages; STING, stimulator of interferon genes; IL-7, interleukin-7; IL-12, interleukin-12; CTLA-4, cytotoxic T-lymphocyte-associated protein 4; DOTAP, 1,2-dioleoyl-3-trimethylammonium-propane.

To overcome these challenges, much attention has been paid to developing delivery methods based on the use of specific surface receptors of immune cells through antibody and receptor binding ligands and other affinity targeting methods. Active-targeting methods have greatly increased the ability to selectively deliver nucleic acid and protein therapeutics to T cells, NK cells, dendritic cells, and macrophages. However, heterogeneity of receptor expression, kinetics of receptor internalization, and off-target binding still affect the efficiency of the targeted delivery process. Recent improvements in LNP technology have significantly enhanced the ability of the LNPs to deliver the genetic material to specific cell populations *in vivo*. To selectively target the CD8 receptor expressed on cytotoxic T cells, Hunter et al. formulated CD8-targeted L829 tLNPs (CD8-L829-tLNPs) for delivery of anti-CD19 or anti-CD20 CAR mRNA. Dosing humanized mouse models with CD8-L829-tLNPs at 30 µg generated quick CAR-T cell activity with complete tumor ablation and continuous depletion of B cells following multiple dosing. To evaluate the system in cynomolgus monkeys, doses from 0.1 to 2.0 mg/kg were administered to demonstrate significant yet reversible B cell depletion and then re-population with naive B cells. Since the system is based on mRNA technology, it offers transient expression of CARs, which may increase safety and control, although repeated administration may be required to maintain therapeutic effects (Hunter et al., 2025). The modified CD8-L829-tLNP formulation facilitated efficient transfection of the cells and consequently led to B-cell depletion and tumor destruction in humanized mice. CAR-T cells derived from PBMCs isolated from the peripheral blood of patients with autoimmune diseases were able to eradicate B cells efficiently showing preserved functionality. In cynomolgus monkeys, the use of tLNPs led to profound but reversible B-cell depletion and subsequently to reconstitution of naïve B cells which is an indication of the restoration of the immunological equilibrium. Overall, this work provides evidence for an alternative therapeutic approach for B-cell tumors and autoimmune diseases (Nyberg et al., 2026). Apart from transient delivery of mRNAs, Bimbo et al. devised an NCtx platform, which is a dual CD3/CD7-targeted LNP technology capable of delivering minicircle DNA encoding CAR and SB100x transposase mRNA in T cells, allowing for stable integration of the CAR sequence into the genome. The one-time intravenous administration at a dose of nucleic acids of 0.05 mg/kg resulted in the generation of persistent CAR-T cells in humanized leukemia models, which showed sustained expression of CAR, specific cytotoxicity, tumor reduction, and increased survival. Furthermore, the platform was able to produce dual CD19/CD22-targeted CAR-T cells with functional persistence. Nevertheless, since NCtx utilizes permanent genomic integration by transposase mechanism, the thorough characterization of integration sites, risk of insertional mutagenesis, and genomic safety needs to be investigated before clinical application (Bimbo et al., 2025).

Nanoparticle surface functionalization using antibody-based capture technology has also been used as a method to increase the specificity of immune-cell targeting. The work by Chen et al. describes the design of an antibody-capturing nanobody-based platform utilizing the TP1107 anti-Fc nanobody immobilized on mRNA-loaded LNPs for capturing targeting antibodies in their optimal orientation, which eliminates the need for modification of antibodies. Anti-CD3, anti-CD4, anti-CD5, and anti-CD7 antibodies were studied for the T-cell targeting effect, and CD3 was selected as the most efficient receptor for the mRNA delivery to human primary T cells. The optimized antibody-capture LNPs were able to deliver the protein with more than 1,000-fold higher efficiency as compared to the non-targeted LNPs and more than eightfold increase in delivery as compared to the conventional methods of antibody functionalization. The intravenous injection of the CD3-targeted LNPs (0.1 mg/kg mRNA in mice) resulted in the transfection of about 50% of splenic CD4^+^ T cells and 54% of splenic CD8^+^ T cells with low delivery to other immune-cell populations (Chen M. Z. et al., 2025). Rational design of nanoparticles is another strategy that contributed to the advancements in immune cell-targeting genome editing. In addition to receptor-mediated targeting, organ-level biodistribution can be exploited to increase immune-cell delivery. Lu et al. identified the spleen-tropic F48 LNP, which preferentially accumulated in the spleen and enabled CRISPR-RNP delivery to splenic T cells. Following intravenous administration of 50 µg of RNP-loaded LNPs, F48 preferentially edited splenic T cells, with T cells accounting for the majority of edited immune cells. Functional studies demonstrated substantial reduction of PD-1 expression, including approximately 70% reduction in total CD3^+^ T cells and approximately 85% reduction in CD8^+^ T cells following PD-1-targeted editing. In a B16-OVA melanoma model, PD-1 RNP delivery also suppressed tumor growth. Although these findings demonstrate efficient spleen-directed T-cell editing, the platform remains predominantly spleen-tropic and its efficiency and cell specificity may depend strongly on LNP composition and the intracellular environment of different immune-cell populations (Lu et al., 2025).

Delivery systems that deliver antigens to professional antigen presenting cells have also been promising for augmenting antitumor immune response. To design a delivery system that can deliver mRNA specifically to antigen presenting cells after systemic administration, For antigen-presenting-cell targeting, Van der Jeught et al. functionalized mRNA lipopolyplexes with a trimannose sugar-tree ligand to promote preferential delivery to dendritic cells following systemic administration. Such lipopolyplexes generate strong antigen-specific CD8^+^ T cell responses along with tumor immunity but are less dependent on Type I interferon signaling than standard mRNA lipoplexes. Notably, the use of mRNA with modifications of N1-methylpseudouridine leads to a decrease in inflammation of cytokines but not to an impairment of T-cell immunity. Nevertheless, the strategy does not promote direct modification of effector T cells and, therefore, relies on indirect tumor immunity mediated by dendritic cells (Van der Jeught et al., 2018).

Collectively, it is clear that specificity depends on biodistribution at an organ level as well as binding to cell surface receptors. The appropriate selection of immune cell targets, along with the fine-tuning of physical and chemical parameters of nanoparticles, is critical to the efficient delivery to the targeted immune cells.

### 2.2 Cellular uptake and intracellular trafficking

After the selective identification of immune cells by specific carriers, the carriers should efficiently be internalized into the target cells and trafficked intracellularly to reach their correct subcellular destination within the cell. Most nanoparticle- and peptide-based drug delivery vehicles are internalized by different mechanisms of endocytosis, including clathrin-mediated endocytosis, caveolae-mediated endocytosis, and micropinocytosis (Maithania and Gundeti, 2026). The specific mechanism used for the internalization of a particular drug delivery vehicle varies based on its physicochemical features, the type of the targeting ligand, and the immune cell type. It is worth noting that different immune cell types have diverse endocytic activities. Specifically, the endocytic activity of resting T lymphocytes is relatively low compared to other professional antigen-presenting cells, like dendritic cells and macrophages (Gao B. et al., 2026). The transported drugs in the endosomes of the cells are trafficked in the early and late endosomes, where the lysosomal degradation significantly hinders the availability of the drugs in the cytosol (Chahal et al., 2026). Some of the approaches considered in literature involve avoiding normal endosomal traffic for the better transport inside the cell. According to Lian et al., there is the development of dithiolane-conjugated LNPs exploiting thiol-mediated endocytosis to enhance the delivery of mRNA inside the cells. Compared with conventional liposomes, the novel formulation provides an increased amount of mRNA transfection by more than 11 times and increases protein expression by 4.5 times compared with liposome-based formulations. Thiol-mediated endocytosis is effective in increasing cytoplasmic delivery without the involvement of traditional endocytic trafficking pathways. However, further studies are required for the understanding of the specificity and safety of this process *in vivo* (Lian et al., 2025). Systematic optimization of nanoparticles has also contributed to efficient intracellular transport of genome-editing cargo. By applying a multistep high-throughput screening procedure, Lu et al. successfully developed a spleen-tropic lipid nanoparticle that could deliver CRISPR-associated proteins to primary T cells. With the use of machine learning in the analysis, important physiochemical characteristics that control nanoparticle uptake, intracellular transport, and genome editing were identified (Lu et al., 2025).

Receptor-mediated internalization is another effective strategy for enhancing intracellular delivery. For example, Chen et al. developed an Fc-binding nanobody-based antibody-capture platform that enabled site-specific functionalization of LNPs with targeting antibodies, resulting in efficient and highly selective mRNA delivery to primary T cells (Chen M. Z. et al., 2025). Besides the engineering of nanoparticles for delivery, quantifying intracellular trafficking has become increasingly significant to determine the determinants of effective delivery. In the study performed by Cavegn et al., a quantitative live-cell imaging system has been developed that uses galectin and Rab protein as reporters for tracking nanoparticle uptake, endosomal escape, recycling, and lysosomal traffic. It was found that there were distinct differences depending on the type of nanoparticle formulation used; thus, LNPs with SM-102 demonstrated 25% endosomal escape, while those with ALC-0315 showed 10%–15% escape along with 7% recycling, both. This information implies that even if the formulations of LNPs look alike, they still may have significant distinctions in the process of intracellular traffic and that cellular uptake does not always reflect cytosolic delivery. One problem with this approach is that the values of traffic were determined under specific conditions (Cavegn et al., 2025). Furthermore, the delivery of the nanoparticles into T-cells does not always require an antibody-mediated targeting. For instance, Fernandes et al. showed that antigen-free, positively charged glycogen nanoparticles can bind human T cells through multivalent physicochemical binding. Both PGEDA and BGEDA nanoparticles showed over 90% binding to human T cells; however, BGEDA, being smaller in size, showed complete cellular internalization, more effective endosomal escape, and about two times more mRNA transfection compared to PGEDA nanoparticles. This data indicates that nanoparticle size, charge, and intracellular transport play an important role in T cell transfection without targeting. Still, due to the fact that the above approach is based on physicochemical interaction and does not involve T cell-specific targeting, it suffers from a major translational drawback (Fernandes et al., 2024). However, the difficulty involved in the intracellular transport of NK cells is of even greater importance because these cells have intrinsic resistance to genetic engineering. The development of DOTAP-labeled dual-functional LNPs was performed by Shin et al. in order to overcome the inherent resistance of NK cells towards genetic modification. The particles showed preferential uptake through clathrin-mediated endocytosis in NK cells and were shown to facilitate the efficient delivery of CAR mRNA to NK cells, while simultaneously triggering the activation of the ERK/MAPK pathway and alteration in mitochondrial dynamics in order to increase the NK cell cytotoxicity. The delivery of glypican-3-directed CAR mRNA resulted in functional CAR-NK cells showing high therapeutic efficacy in the orthotopic liver cancer mouse model. However, preclinical studies have been performed to validate this approach mainly on NK cells (Shin et al., 2024). In the same vein, intracellular trafficking has been revealed to be an important consideration for the engineering of macrophages. PSβ-LNPs were prepared through the addition of phosphatidylserine as the macrophage-recognition group along with β-sitosterol, which helped to enhance the mRNA transfection efficacy. Through an experiment using the CT26 peritoneal metastasis mouse model, after a single intraperitoneal injection, there was GFP expression in over 60% of the peritoneal macrophages after 18 h, whereby macrophages made up over 80% of the cells that underwent transfection. About 23% of the intratumoral F4/80^+^ macrophages were transfected too. The PSβ-LNPs were able to mediate the *in-situ* development of CAR macrophages and tumor immune microenvironment reconstruction. However, CAR expression was transient and decreased significantly with continuous antigen stimulation (Gu et al., 2025).

Collectively, these studies indicate that successful *in vivo* immune-cell engineering requires coordinated optimization of multiple delivery steps rather than improvement of a single parameter. Selective immune-cell targeting must be coupled with efficient cellular uptake, intracellular trafficking, endosomal escape, and functional cargo release, as increased uptake alone may not translate into greater biological activity when cytosolic delivery remains inefficient. Thus, achieving an optimal balance among biodistribution, cell specificity, intracellular transport, and cargo release represents a central design principle for next-generation delivery systems. Further elucidation of the molecular mechanisms governing cellular uptake, endosomal trafficking, membrane fusion, and cytosolic release will be critical for improving delivery efficiency and therapeutic performance.

### 2.3 Endosomal escape and payload release

Once taken up by the cell, one of the major challenges of efficient intracellular delivery of therapeutic molecules is endosomal sequestration, which greatly hampers the bioavailability of the delivered cargoes. Despite the high efficiency of nanoparticle uptake through different routes of endocytosis, most of the loaded nucleic acids get entrapped in early or late endosomes, which later become degraded inside lysosomes (Mazumdar et al., 2021). This poses a problem for intracellular therapies like mRNA, siRNA, CRISPR/Cas genome editing, and other nucleic acid-based treatments, as these depend upon reaching the cytoplasm or nucleus to act on the target cells (Singh et al., 2026).

To address this problem, much research has been conducted with the aim of designing vehicles which will be capable of efficient endosomal disruption or fusion. Ionizable lipids, cell-penetrating peptides, fusogenic lipids, acid-sensitive polymers and stimuli-responsive biomaterials have been synthesized to disrupt the endosomal membrane in an acidic environment to enable the release of the drug into the cytosol while causing no toxic effect on the cells (Balenzano et al., 2025). The second thing that should be considered is the controlled release of drugs from the delivery vehicle after the endosomal escape since the insufficient drug release will affect its biological activity (Pei, 2025).

LNPs recently developed through lipid nanoparticle engineering research have led to the elucidation of important mechanisms underlying endosomal escape. Jozić et al. synthesized branched ionizable phospholipids and found BiP-20 to be highly efficient as a LNP delivery system for mRNA and CRISPR-based genome editing. BiP-20 demonstrated about eightfold higher CRISPR-Cas9 efficiency compared to control LP01 at low concentrations. Live-cell imaging with LysoBC/LysoTag showed that about 8% of BiP-20 LNPs were able to escape from endosomes after 30 min of treatment. It was further found that late-endosome maturation is a limiting step since the inhibition of Rab7 significantly improved endosomal escape. However, the optimization was conducted primarily for liver cells; therefore, it still needs to be determined whether the same advantages will be preserved in immune cells such as T-cells and NK-cells (Jozić et al., 2026).

Various nanoparticle structures have been created for enhancing endosomal escape using physicochemical optimization. Li et al. developed Au-core LNPs in which an engineered gold-containing core reorganizes the internal architecture of the nanoparticle to enhance pH-responsive endosomal membrane disruption. Compared with conventional LNPs, Au-LNPs achieved approximately twofold greater endosomal escape and nearly 100-fold greater cytoplasmic mRNA diffusion. At low mRNA concentrations, Au-LNPs produced approximately 91.4% cellular transfection compared with 6.72% for conventional LNPs, and *in vivo* mRNA expression increased by as much as sevenfold depending on the administration route. In a WT1 mRNA vaccine model of triple-negative breast cancer, Au-LNP treatment reduced tumor size and tumor weight by approximately 87% and 86%, respectively, compared with PBS. Although these findings demonstrate the importance of nanoparticle internal architecture, the use of a gold core represents a proof-of-concept strategy, and future translation will likely require biodegradable or clinically established core materials (Li T. et al., 2026).

In addition to the conventional ionizable lipids, a group of structurally modified lipid derivatives have also emerged as potent vehicles for facilitating intracellular cargo release. In a recent study by Habrant et al., ionizable lipids based on tobramycin were designed for increased delivery of nucleic acid drugs and endosomal escape of therapeutic nucleic acids. Of the several lipids formulated, one was found to have the best efficiency of delivering mRNA, plasmid DNA, and siRNA–this lipid was designated as 30 and is known to be a DOSST lipid. Using cryo-transmission electron microscopy, it was noted that DOSST lipids can form small and homogeneous nucleic acid complexes compared to other formulations; thus, making transport of these complexes into the cells easy. The high efficacy of DOSST lipids could be due to their efficient mixing with membranes (Habrant et al., 2016).

In summary, all these studies indicate that the ability to escape from endosomes is still one of the main drivers behind efficient intracellular delivery of nucleic acid-based therapeutics. Future advancements in ionizable lipids, nanocarrier design, active membrane-binding biomaterials, and knowledge about intracellular trafficking mechanisms would likely contribute to better intracellular delivery performance while decreasing inflammation and intracellular degradation of therapeutic compounds. These breakthroughs would be vital in optimizing *in vivo* immune cell engineering and clinical translation of novel immunotherapy approaches using RNA and gene editing. The major biological barriers, targeted delivery platforms, and the overall workflow of *in vivo* immune cell engineering are summarized in Figure 2.

**FIGURE 2:**
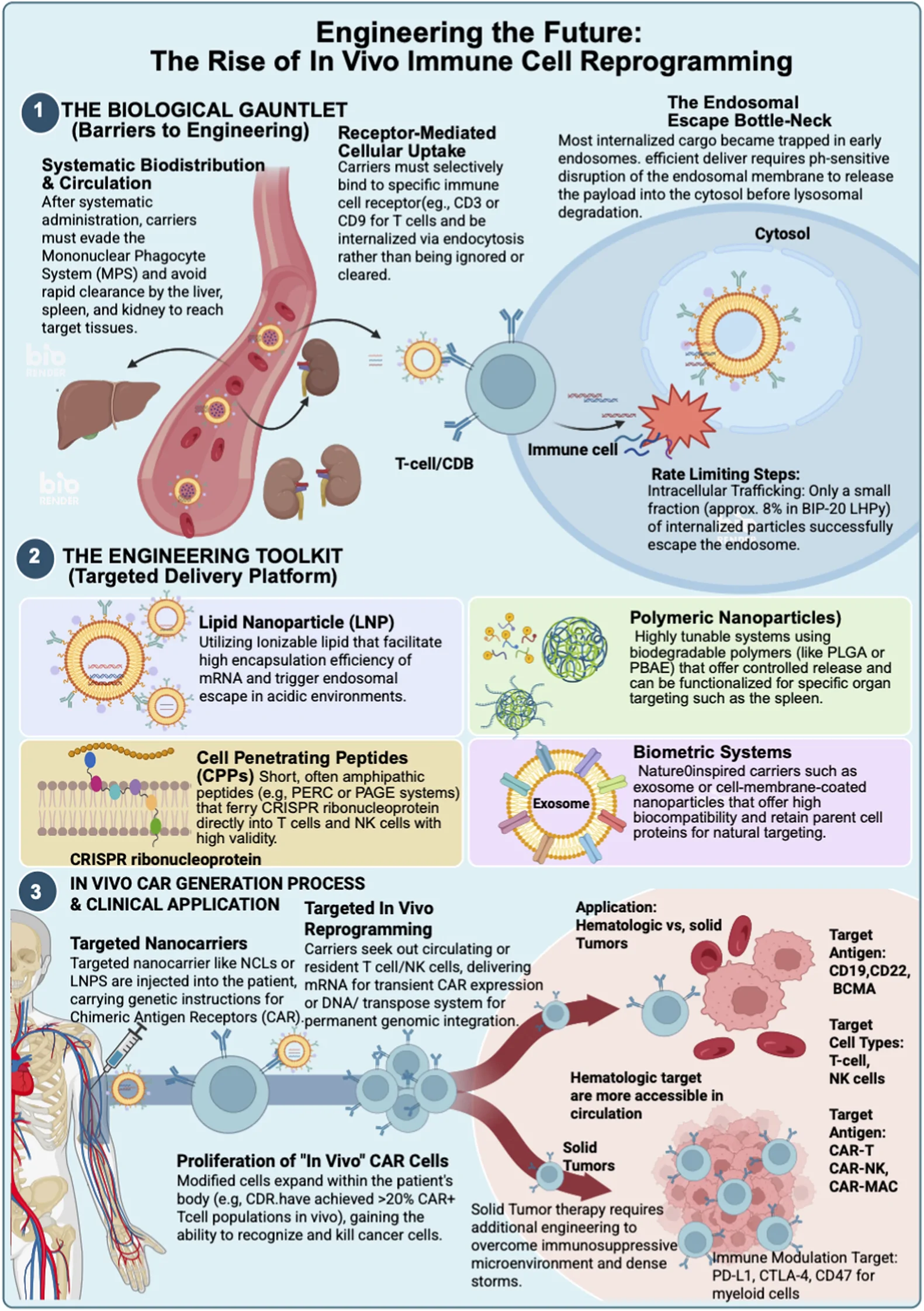
Engineering the future: targeted platforms and challenges for *in vivo* immune cell reprogramming. Schematic representation of the major biological barriers, delivery technologies, and therapeutic applications of *in vivo* immune-cell engineering. (1) Biological barriers: Following systemic administration, delivery systems must achieve favorable biodistribution, avoid rapid clearance by the mononuclear phagocyte system, selectively recognize immune-cell receptors, undergo efficient cellular internalization, and escape endosomal sequestration to release therapeutic cargo into the cytosol. Endosomal escape remains a major rate-limiting step, as only a small fraction of internalized nanoparticles successfully reach the cytoplasm. (2) Targeted delivery platforms: LNPs, polymeric nanoparticles, CPPs, and biomimetic systems such as exosomes and cell-membrane-coated nanoparticles can deliver nucleic acids, proteins, and gene-editing components to specific immune-cell populations. (3) *In vivo* engineering and therapeutic application: Systemically administered targeted nanocarriers can directly reprogram endogenous T cells, NK cells, or myeloid cells, enabling their expansion and antitumor activity without conventional *ex vivo* manufacturing. Engineered immune cells can target established antigens such as CD19, CD22, and BCMA in hematologic malignancies, whereas applications in solid tumors include CAR-T, CAR-NK, and CAR-macrophage strategies combined with approaches targeting immunosuppressive pathways such as PD-1/PD-L1, CTLA-4, and CD47. Together, these advances illustrate the potential of targeted delivery technologies to enable scalable and precise *in vivo* immune-cell engineering for cancer immunotherapy. Created with BioRender.

## 3 Delivery platforms for *in vivo* immune cell engineering

The efficacy of immune cell modification *in vivo* is highly dependent on the design of delivery systems which would be able to deliver therapeutic cargos specifically and efficiently into desired populations of immune cells. As opposed to standard delivery systems, delivery systems which specifically deliver their contents to immune cells have to overcome a set of challenges that include long-term systemic circulation, specific cell targeting, efficient intracellular transport, regulated cargo release, low off-target toxicity, and clinical-scale manufacturability (Sun et al., 2026). Significant advances in biomaterial science, nanotechnology, and molecular targeting have made possible the creation of numerous delivery systems designed to address these issues. Some of the most advanced techniques include cell-penetrating peptides (CPPs), antibody and receptor-based targeting systems, and nanoparticle delivery systems (Liu Z. et al., 2026). The major targeted delivery platforms currently used for *in vivo* immune cell engineering are summarized in Table 1.

### 3.1 Cell-penetrating peptides

CCPs are highly versatile short peptides that are either positively charged or amphipathic in nature and can carry different cargo into the cells. The capacity of CPPs to ferry different cargo such as nucleic acids, proteins, peptides, ribonucleoprotein particles, and genome editors is promising for immune-cell engineering since many types of immune cells such as resting T-cells and NK cells have poor transfection efficiencies via conventional methods (Patel et al., 2024). Depending on their structure, the mechanism by which CPPs transport their cargo can be by direct translocation into the cell membranes or endocytosis, thus allowing for delivery to target cells and overcoming the problems associated with viral vectors. In addition to the flexibility in the cargo being transported, the following features of CPPs make them ideal tools for transient transfection, genome editing, and cell reprogramming: easy synthesis, modular design, and conjugation to various therapeutic agents (Di Pisa et al., 2015). However, one of the major limitations of traditional CPPs includes low cell specificity, fast degradation, non-specific distribution in the body and inefficient release from the endosomes after entering the cells. These factors greatly affect the efficiency of CPPs following systemic application. Therefore, the current efforts in the development of new CPPs are directed towards improving stability, specificity, and intracellular trafficking abilities (Wang H. et al., 2026). These recent innovations have shown that CPPs can be used to efficiently deliver genes into primary immune cells. For instance, Foss et al. reported Peptide-Enabled RNP Delivery for CRISPR Engineering (PERC), which is an amphiphilic A5K-based CPP system for delivering CRISPR ribonucleoprotein complexes to primary human lymphocytes. As compared to electroporation, PERC resulted in efficient gene editing in primary T cells, B cells, and NK cells with improved cell viability and limited alteration of the transcriptome. Furthermore, the technology enabled multiplex gene editing, adenine base editing, and specific integration of the CAR into the TRAC locus, which produced greater yields of CAR-T cells with anti-tumor activities. Notably, the sequential delivery of peptides resulted in minimal chromosomal translocations and maintenance of naïve T-cell phenotypes (Foss et al., 2023). In the same vein, Zhang et al. created the Peptide-Assisted Genome Editing (PAGE) technology which is another CPP delivery system comprising a combination of CPPs-conjugated Cas9 or Cas12a ribonucleoproteins and an optimized endosomal escape peptide to increase the intracellular genome editing efficiency. PAGE was able to edit the genome with up to 98% efficiency in human and murine T cells as well as 100% efficiency in human hematopoietic stem and progenitor cells within a short incubation time. Unlike electroporation, PAGE caused little toxicity, preserved global transcription profiles, allowed multiplex editing and CAR-T cell expansion and function (Zhang et al., 2024).

Taken together, these papers show how the recent progress in CPP engineering has greatly enhanced the possibilities of utilizing peptide carriers in delivering cargo to manipulate immune cells. Further development of peptides in terms of their structure, selectivity of delivery, intracellular trafficking, and endosomal escape is likely to render even more effective clinical application of such delivery approach.

### 3.2 Antibody and receptor-mediated targeting

Receptor- and antibody-mediated targeting have been found among the most efficient ways of delivery of drug-loaded nanoparticles to the immune cells. Such techniques make use of the presence of the surface receptors which are expressed on the surfaces of some immune cells but not on others. Monoclonal antibodies, antibody fragments, bispecific antibodies, receptor-binding ligands, and peptides have been studied as targeting agents capable of delivering nanoparticles, DNA, RNA, proteins, and other cargoes to particular immune cells (Singh et al., 2019). As far as the delivery of various molecules to T-cells is concerned, the receptors such as CD3, CD4, CD5, CD8, and CD90 receptors were extensively studied due to their specific expression and good receptor-mediated uptake into the cells. Also, NK cell receptors including NKG2D, NKp46, and CD16 can be used for the delivery to types of NK-cells. Receptor-mediated delivery system differs from passive delivery systems in terms of its higher efficiency and reduced side effects due to its increased cellular uptake, improved intracellular trafficking, and less exposure to systemic circulation. Also, receptor-binding peptides and ligands are considered more promising than antibodies since they are much more immunogenic, penetrate deeper into tissues, and are easy to synthesize and store (Page et al., 2024). Further research has highlighted the efficacy of receptor-directed immune cell targeting. Lutz et al. developed a range of bispecific antibodies directed towards NKG2D receptors by incorporating human single chain variable fragments against NKG2D with antibodies targeting CD20 to increase the immune mediated clearance of B-cell malignancies. The bispecific antibodies generated were shown to activate NK cells, kill lymphoma cells directly and improve antibody dependent cellular cytotoxicity when co-administered with therapeutic antibodies. While there was not much in terms of CD8^+^ T cell activation, combination with a CD19 × CD3 bispecific T-cell engager led to increased T cell mediated cytotoxicity (Lutz et al., 2023).

Targeting NKG2D ligands has been demonstrated to be useful in inducing enhanced immunity against solid tumors. Hagelstein et al. generated NKG2D-CD3 and NKG2D-CD16 fusion proteins that are specific for NKG2D ligand-bearing soft tissue sarcomas and that recruit T-cells and NK cells at the same time. NKG2D-CD3 fusion protein triggered strong activation of T cells, release of cytokines and degranulation with significant cell death of tumor cells, but on the other hand, the NKG2D-CD16 fusion protein was able to trigger NK cells and cause fast cytotoxic reactions. Both NKG2D-CD3 and NKG2D-CD16 fusion proteins were still potent in inducing antitumor response against patient-derived sarcomas, even when immune cells from heavily treated patients were utilized (Hagelstein et al., 2021). This idea was extended further by Zekri et al., who have engineered a modified B7-H3×CD3 IgG bispecific antibody termed CC-3 to direct T cells against B7-H3 expressing gastrointestinal cancers. The modified CC-3 directed to a membrane proximal B7-H3 epitope and decreased the affinity for CD3 not only preserved efficient T cell activation, proliferation, memory generation, and killing of target cells but also minimized the cytokine production related to systemic toxicity. In various *in vivo* experiments, CC-3 displayed long-term serum half-life, significantly inhibited colorectal cancer growth, prevented lung metastasis, eradicated tumors, and did not trigger the activation of non-specific T cells (Zekri et al., 2023).

In conclusion, no single delivery platform can satisfy all criteria associated with *in vivo* immune engineering. As far as non-viral delivery modalities go, currently available LNPs are the best compromise between effective nucleic acid delivery, manufacturing scalability, modularity of targeting and repeat dosing ability, whereas polymeric nanoparticles are better at controlling degradation and release of the payload. CPP delivery is highly effective in terms of intracellular delivery and genome editing, although more work needs to be done to improve cell specificity of such delivery platforms for *in vivo* use. Biomimetic delivery systems show high biocompatibility and intact biological activity of the system, however, manufacturing and scaling up of such systems is problematic. Antibodies and receptors serve as modular components used to increase cell specificity in these systems. Viral vectors include AAV which shows efficient and persistent expression of transgene, however, have certain limitations associated with packaging limitations, induction of neutralizing antibodies and inability to be dosed repeatedly; lentiviral vectors show efficient genome integration and stable expression, however, the use of these vectors is associated with manufacturing complexity and safety concerns regarding insertional mutagenesis. *Ex vivo* CAR-T cell therapy is the established approach to cell therapy, which uses controlled cell manipulation and careful quality assurance before injection into the patient, however, requires customized manufacturing and long production time. Therefore, platform choice depends on target immune cells, cargo, required time of gene expression, possibility of repeat dosing, manufacturing abilities and safety issues.

## 4 Nanoparticle-based systems

Delivery platforms that employ nanoparticles have gained traction as a key aspect of *in vivo* immune cell engineering due to the superior performance in protecting therapeutic payloads from enzymatic degradation, extending the circulation time of particles, and enabling targeted delivery to immune cells. Nanoparticles provide an impressive level of flexibility when it comes to chemical composition, particle size, surface chemistry, and ability to carry certain loads compared to traditional approaches to delivery (Ji et al., 2025). Moreover, the employment of targeting molecules, stimuli-sensitive materials, and specific surface chemistry tailored for immune cells has led to enhanced delivery specificity and low levels of systemic toxicity. New developments in nanotechnology have led to the use of lipid, polymeric, and biomimetic nanoparticles for delivery of mRNA, siRNA, plasmids, proteins, and genome editing tools, and these platforms have been successfully employed as alternatives to viral delivery of genetic material to immune cells (Bhairam et al., 2026).

### 4.1 Lipid nanoparticles

LNPs are considered the most clinically advanced class of non-viral delivery systems for nucleic acid medicines and are gaining more attention as one of the essential enabling technologies for *in vivo* immune cell engineering. Traditional LNPs are usually formed using a combination of ionizable lipids, phospholipids, cholesterol, and PEG-lipids, which together provide effective encapsulation and delivery to cells of various therapeutic cargoes such as mRNA, siRNA, plasmid DNA, and genome editors (Kambanis et al., 2026). Successful use of mRNA vaccines via LNPs has stimulated the development of specialized LNPs that allow selective reprogramming of T cells, NK cells, dendritic cells, and other immune cells *in vivo*. Advances in lipid chemistry, surface modification, and targeted delivery to specific organs have significantly enhanced the delivery specificity of LNPs and minimized adverse effects (Roy et al., 2026).

One approach that can enhance specificity in immune cell targeting is the use of antibody-mediated surface modification of LNPs. Using a method that involves conjugation of antibodies specific to pan-T-cell markers (CD3, CD5, and CD7) on C14-4 LNPs, Billingsley et al. developed antibody-functionalized ionizable LNPs (Ab-LNPs). This approach enabled redirection of biodistribution of the particles from the liver to lymphoid organs. Among all the evaluated LNPs, the CD3-targeted Ab-LNPs showed high transfection efficiency and resulted in the formation of transient but functional CAR-T cells specific for CD19 and capable of eliminating around 90% of the B cells after intravenous injection. In particular, the dose-dependent production of cytokines and low toxicity make the antibody-mediated LNPs a promising scalable alternative to the viral vectors used to produce CAR-T cells *in vivo* (Billingsley et al., 2024). In addition, selective targeting of receptors has been coupled with immune-modulating cargo loading to increase the therapeutic efficiency of the treatment. For example, Li et al. designed CD3-targeted ionizable LNPs to allow direct *in situ* engineering of CAR-T cells by using targeted delivery of CAR mRNA to be circulating T cells. The use of anti-CD3 antibody for LNPs was shown to lead to efficient splenic localization of the particles as well as to reduce off-target gene expression. Moreover, co-delivery of IL-7 mRNA resulted in IL-7 secreting CAR-T cells exhibiting better proliferation, persistence, central memory formation, and cytotoxic activity. It was demonstrated that in a model of murine melanoma, such approach led to suppression of tumor growth, recruitment of functional CD8^+^ T cells, as well as better efficacy in combination with PD-1 blockade without inducing cytokine release syndrome and systemic toxicity (Li T. et al., 2024). While there has been a lot of emphasis on the direct engineering of immune cells *in vivo*, LNPs have shown great promise in terms of transient *ex vivo* engineering of lymphocytes for therapeutic purposes. In a comparative study conducted by Kitte et al., the optimized LNP mediated delivery of mRNAs was found to be more effective in delivering the CAR mRNA for the generation of CAR-T cells specific for CD123 than the traditional electroporation approach. This was because LNP mediated transfection increased the duration of presence of intracellular CAR mRNA, enhanced cell viability, decreased T-cell exhaustion, and sustained surface CAR expression. The engineered CAR-T cells were found to have high cytotoxic effects with reduced off target effects (Kitte et al., 2023).

In recent studies, it has been shown that the use of LNP-based cell delivery can achieve sustained immune cell engineering without any *ex vivo* cell manipulation. In one study, Zhou et al. created an LNP-based CD3-targeting platform to co-deliver both a plasmid containing CD19 specific CAR genes and shRNA against IL-6. This led to highly effective targeted lymphoid delivery and formation of CAR-T cells that expressed CAR for at least 90 days and had potent antileukemia effects that were like *ex vivo* CAR-T cells. It was additionally noted that concomitant inhibition of IL-6 substantially reduced inflammation associated with cytokine release syndrome without affecting therapeutic potential (Zhou et al., 2022). In addition to mRNA-based transient expression, the ability to achieve permanent genetic modifications has also recently been realized by means of non-viral genome editing technologies. Bimbo et al. designed a dual CD3/CD7 targeting LNPs platform called NCtx that delivered a combination of minicircle DNA harboring CAR sequences along with mRNA coding for SB100x transposase, thus ensuring stable integration after systemically administering this combination. The efficient delivery to both resting and activated T cells was observed, resulting in the stable expression of the CARs, durable cytotoxic activity against antigens, and tumor regression upon just one intravenous injection in a humanized model of leukemia. Moreover, this platform allowed producing dual-targeting CD19/CD22 CAR-T cells that demonstrated prolonged persistence and memory phenotypes with very limited T cell exhaustion (Bimbo et al., 2025). Besides achieving cell specificity, there is an increasing interest in solving the inherent drawbacks associated with the existing LNP formulations including liver deposition and PEG-related immunogenicity. These issues were overcome by Zhao et al., who developed cholesterol and PEG-free three-component LNPs (ThrCo LNP) in which zwitterionic PyCB ionizable lipids replaced both cholesterol and PEGylated lipids. The rationally designed delivery system showed 4.5-fold higher mRNA translation in the spleen with the reduction in the hepatic accumulation by about 70% compared with clinical LNP formulation of BNT162b2. ThrCo LNPs were shown to deliver the mRNA specifically to spleen dendritic cells and macrophages resulting in potent antigen-specific CD8^+^ T cell response and immunological memory formation. Notably, the delivery system preserved its effectiveness upon repetitive delivery by decreasing protein adsorption and preventing accelerated blood clearance. In melanoma models, the use of mRNA vaccines based on ThrCo LNP suppressed the primary tumor growth, lung metastasis, and recurrence, prolonging overall survival (Zhao et al., 2025b).

A trajectory for emergent design involves the evolution of passive nucleic acid delivery into programming for immune engineering that targets specific cells and organs. The latest trends in LNP development involve the convergence of all four features mentioned above in one delivery system. It is particularly important to highlight the fact that the temporary delivery of mRNA can be more suitable for reversible activation of immune cells, while DNA delivery or genome editing can be used for permanent reprogramming.

### 4.2 Polymeric nanoparticles

The advent of polymeric nanoparticles as a unique and multifunctional type of non-viral delivery systems is linked to their outstanding tunability in terms of physicochemical properties, high loading capacity, and excellent biocompatibility. Contrary to the lipid carriers, polymeric nanocarriers may easily be tailored to ensure fine regulation of particle sizes, surface charges, degradation rates, and controlled cargo delivery profiles offering considerable possibilities for nucleic acids, proteins, peptides, and immunomodulators delivery (Austria et al., 2025). Numerous studies have been conducted on application of biodegradable polymers such as PLGA, poly (β-amino ester) (PBAE), PEI, and hydrogel-based materials for delivery into immune cells. Moreover, functionalization of nanocarriers with targeting ligands, stimuli-responsive polymers, and membrane disruptive elements have enhanced cellular uptake, intracellular transport, and endosomal escape thus improving their therapeutic properties (Ahmed et al., 2026). The remodeling of the immune environment of tumors is among the key areas of application of polymeric nanovesicles. Yuan et al. formulated 2-hydroxypropyl-β-cyclodextrin (2-HP-β-CD) modified PLGA nanoparticles loaded with TLR7/8 agonist R848 to selectively target TAMs. The nanoparticles had favorable physicochemical properties such as high drug loading efficiency, pH-dependent drug release and efficient macrophage uptake. They resulted in effective conversion of M2 macrophages that exhibit an immunosuppressive role in tumor into pro-inflammatory M1 macrophages. In the treatment of MC38 colon carcinoma along with anti-TNFR2 therapy, there was increased tumor accumulation, CD8^+^ T cell infiltration, pro-inflammatory cytokine production, decreased regulatory T cell populations, suppression of tumor growth, increased survival and development of durable immune memory in the absence of systemic toxicity (Yuan et al., 2024).

Polymers were further used in designing delivery systems for efficient delivery of nucleic acids into secondary lymphoid organs. Zhang et al. created the fluorinated ionizable nanoparticle SSC6F5, which was identified through high throughput screening among 312 fluorinated ionizable lipids for selective mRNA delivery in the spleen. SSC6F5 showed significantly better splenic specificity than the clinical SM102 LNPs and SORT formulations in addition to better mRNA transfection after intravenous administration. This is because of the formation of a protein corona rich in apolipoprotein D (Apod) that enabled efficient targeting of splenic immune cells, efficient escape from the lysosome, and efficient mRNA translation within the cells. Consequently, the delivery system efficiently achieved efficient genome editing of macrophages, dendritic cells, T cells, and B cells, and activated effective cell-mediated and humoral immunity against tumor antigens in melanoma models. Consequently, the antitumor immunity was significantly increased to inhibit tumor growth and prolong life span with excellent biosafety (Zhang Y. et al., 2025). Antigen presentation for prolonged periods has emerged as an additional method of boosting antitumor immunity. In a study carried out by Wang et al., a patient’s personalized hydrogel vaccine was designed using immunogenic cell death-induced autologous tumor antigens in combination with MSA-2 STING agonist to ensure prolonged immune activation. The vaccine used gallic acid-manganese-mitoxantrone (GMM) nanoparticles to stimulate immunogenic cell death leading to the release of individual patient’s tumor antigens and damage-associated molecular patterns (DAMPs). Subsequently, these immunogenic elements were incorporated in an injectable alginate hydrogel to allow for sustained release of the antigens and the prolonged activation of dendritic cells using the cGAS-STING pathway. When tested on mouse models of melanoma and breast cancer, the personalized vaccine led to the improved maturation of dendritic cells as well as their activation together with CD4^+^ and CD8^+^ T cells. There was also increased population of effector and memory T-cells that successfully suppressed both primary and metastatic tumors. Combined with anti-PD-L1 therapy, the vaccine boosted antitumor immunity through increased numbers of tumor-infiltrating cytotoxic T-cells and depletion of immunosuppressive cells (Wang N. et al., 2026). Systemic delivery of mRNA vaccines using biodegradable polymeric nanoparticles has been demonstrated to be very promising as well. Ben-Ben-Akiva et al. developed biodegradable hydrophobic PBAE nanoparticles that enabled systemic delivery of mRNA to splenic dendritic cells without any need for ligands. R18D nanoparticle systems were found to effectively deliver tumor-antigen coding mRNA along with Toll-like receptor agonists, enabling more efficient transfection of dendritic cells, better endosomal escape, and splenic localization of nanoparticles following intravenous injection. Efficient activation of dendritic cells was shown through upregulation of CD40 and CD86 expression, which led to potent CD8^+^ T-cell response and inhibited the tumor growth in several types of mouse melanomas and colon carcinomas. Moreover, combination with PD-1 checkpoint blockade extended the survival and generated durable immunity against rechallenge with tumors. These results confirm biodegradable PBAE nanoparticles as efficient carriers for systemic mRNA vaccines (Ben-Akiva et al., 2023).

Overall, these research findings confirm that polymeric nanoparticles can be used as highly flexible delivery systems, which can combine targeted drug delivery, sustained antigen delivery, regulated cargo release, and cell-specific modification of immune cells into one system. Further advancements in polymer chemistry, biodegradable polymers, stimuli-responsive systems, and cell-specific immune cell targeting techniques will allow for even more precise and efficient application of polymeric nano-systems in the engineering of *in vivo* immune cells and novel cancer immunotherapy techniques.

### 4.3 Emerging biomimetic systems

Recent developments include the emergence of biomimetic delivery systems, a promising type of nanocarrier that utilizes natural biological structures to improve the efficacy and specificity of delivery. Biomimetic delivery systems such as extracellular vehicles, exosomes, cell membrane-coated nanoparticles, and membrane-derived nanovesicles have many of the features associated with their parent cells, unlike artificial nanoparticles (Guo et al., 2025). Therefore, they have high biocompatibility, long circulation times, low immunogenicity, and the ability to target tissues. Moreover, biomimetic systems can maintain the membrane proteins related to adhesion, immunological recognition, and cell-cell communication of the parent cells, making them able to interact effectively with immune cells while avoiding any other interactions. Even though most biomimetic delivery systems are still in the preclinical development phase, there have been recent breakthroughs highlighting their enormous potential in immune targeting, *in vivo* engineering of immune cells, and cancer immunotherapy (Zhang et al., 2026a).

Among various exosomes produced by immune cells, immune-cell derived extracellular vesicles have been shown to be particularly promising as multi-functional vectors that can deliver antigens as well as immunoregulating molecules at the same time. The team of researchers, headed by Chen, developed dendritic cell derived exosomes (DEV@IL-12-aCTLA-4), which were modified with IL-12 and anti-CTLA-4 antibodies on extracellular vesicles isolated from mature dendritic cells that had been activated with neoantigen, lipopolysaccharide, and interferon-γ. These vesicles retained several important antigen-presenting proteins such as MHC-I, CD80, CD86, and tumor associated antigens, thus maintaining their inherent ability to stimulate T cells. Modification of surface with IL-12 and anti-CTLA-4 provided an additional costimulatory signal and reduced inhibition caused by the action of immune checkpoint and led to powerful stimulation of CD4^+^ and CD8^+^ T cells along with increased IFN-γ production and proliferation of T cells. After subcutaneous injection, these vesicles accumulated in the draining lymph nodes, thus stimulating local immune response with minimal systemic effects. In melanoma and lung carcinoma mice, DEV@IL-12-aCTLA-4 induced a potent Th1-type immune response, revitalized CD8^+^ T cells, effectively suppressed tumor growth, and was highly biologically safe (Chen J. et al., 2025). Membrane-coated nanoparticles are another promising strategy for biomimicry by leveraging both the benefits of artificial nanoparticles and biological characteristics of the cell membrane. Zhao et al. created a nanoparticle with the membrane of the macrophage (M1@PLGA–PEG–TMP195) to deliver TMP195 histone deacetylase inhibitor to tumor-associated macrophages. The membrane coating increased tumor localization, triggered pH-sensitive drug release, and contributed to the effective conversion of immunosuppressive M2 macrophages into M1 macrophages. The remodeling of the tumor microenvironment significantly increased the effect of PD-1 blocking treatment leading to apoptosis of tumor cells, decreased angiogenesis and proliferation of the tumor, and inhibited tumor growth in murine models of breast cancer with no signs of systemic toxicity. Thus, it is possible to conclude that the coating of nanoparticles with the cell membrane increases targeting and remodeling of the immune microenvironment of the tumor (Zhao et al., 2025a).

The latest developments have taken the field of biomimetic engineering even further to utilize immune cell membranes as therapeutic agents. AM-dLNPs were synthesized in the study conducted by Du et al., which involved the coating of STING agonist loaded liposomes using membranes derived from activated T cells. The presence of PD-1, LAG-3, TIM-3 and several adhesion molecules in the membranes provided the capacity to competitively bind and uptake the immune checkpoint ligands along with delivering the STING agonist to the tumor environment. This double-edged strategy improved the activation of dendritic cells, increased the infiltration of T cells within the tumors, increased inflammation associated cytokine secretion, and restored the function of cytotoxic T cells while counteracting immune suppression. In multiple murine models, AM-dLNPs outperformed immune checkpoint blockade and STING agonist therapy separately, showing their potential use as therapeutics through synergistic approach towards cancer immunotherapy (Du et al., 2026). Extracellular vesicles have also shown their high efficiency as vehicles for targeted mRNA delivery. Huo et al. developed LpqH tagged macrovesicles (LpqH-MVs) by incorporating the ectodomain of LpqH that binds to mannose receptors in mycobacteria such as *M. tuberculosis* to the surface of extracellular vesicles. Compared to LNPs and exosome nanoparticles, LpqH-MVs showed greater specificity for macrophages, higher efficiency of mRNA loading, and intracellular mRNA delivery via mannose receptor-mediated internalization. mRNA vaccines delivering mRNA encoding viral antigens stimulated potent humoral and cellular immune responses, producing antigen-specific antibodies and activating IFN-γ secreting CD4^+^ and CD8^+^ T cells. Besides, the developed biomimetic approach resulted in enhanced protection from viral infection with less inflammation and systemic toxicity in comparison to conventional LNPs (Huo et al., 2026). The potential of dendritic cell membrane nanovesicles that mimicked biomimetic properties was explored to improve the presentation of antigens and the activation of the innate immunity. Zhang et al. generated a biomimetic nanovesicle called Ag-DCNV-STINGa by engineering tumor antigen-induced mature dendritic cells into nanovesicles and functionalizing them with a STING agonist named 2′3′-cGAMP. The biomimetic nanovesicles possessed many antigens such as MHC-I, MHC-II, CD11c, and CD86, and thus were able to be effectively taken up by the antigen-presenting cells and activate the STING-TBK1-IRF3 pathway. Innate immunity pathway activation triggered the maturation of dendritic cells, secretion of inflammatory cytokines and chemokines, activation of CD4^+^ and CD8^+^ T cells, and killing of tumor cells. Systemic administration of Ag-DCNV-STINGa in humanized bladder cancer models could effectively inhibit the growth of the primary tumor, reduce the metastasis of tumors in lungs, induce the infiltration of immune cells, tertiary lymphoid structure formation, and maintain good biosafety with low systemic toxicity (Zhang et al., 2026b).

Biomimetic delivery systems can be collectively regarded as a new wave of immune cell-specific nanotechnology combining the biological benefits of natural cell membranes with the versatility of artificial nanomaterials. Their capacity for retaining the native targeting capabilities, ensuring enhanced immune system compatibility, ensuring effective intracellular delivery, and modulation of multiple immune pathways makes them promising platforms for the development of the next-generation of immune cell engineering *in vivo*. Further developments in exosome engineering, membrane functionalization, fabrication processes, and cargo loading techniques will contribute to faster translation of such systems into precision medicine for cancer immunotherapy.

### 4.4 Viral vectors for *in vivo* immune cell engineering

Viral vectors remain unique *in vivo* genetic engineering tools because of their ability to mediate efficient cellular transduction and sustain gene expression. Different adeno-associated viral (AAV) vectors, adenoviral vectors, and lentiviral vectors were considered for the delivery of therapeutic genes or genome editing tools. AAV vectors usually provide prolonged transgene expression with moderate immunogenicity while having small packaging capacity and neutralizing antibodies as limiting factors. Adenoviral vectors are characterized by efficient transduction and higher cargo capacity but may cause strong innate and adaptive immune responses. On the contrary, lentiviral vectors allow delivering relatively big genetic material and stable transgene expression via genomic integration, making them appealing candidates for persistent immune cell reprogramming (Demircan et al., 2024).

The most recent progress in vector development allows achieving selective *in vivo* genome engineering in immune cells. Nyberg et al. used a combination of AAV-mediated delivery of a DNA donor template to T cells and CRISPR–Cas9-loaded enveloped delivery vehicles (EDVs) for *in vivo* site-specific CAR insertion in the TRAC locus, generating functional CAR-T cells and exhibiting antitumor activity in humanized mice models (Nyberg et al., 2026). Andorko et al. created INT2104, an engineered lentiviral vector targeting CD7 for the generation of anti-CD20 CAR-T and CAR-NK cells *in vivo*. Single intravenous injection resulted in generation of CAR-T and NK cells, depletion of B-cells, and elimination of established B-cell tumors in humanized mice, while selective targeting of T and NK cells was shown in cynomolgus macaques (Andorko et al., 2025). Hamilton et al. designed antibodies-targeted Cas9-EDVs for delivery of preassembled CRISPR–Cas9 ribonucleoprotein complexes into selected human T-cell populations. In humanized mice, systemic delivery allowed for *in vivo* generation of CAR-T cells and disruption of TRAC, illustrating potential of virus-derived particles for programmable genome engineering (Hamilton et al., 2024). Hamilton study is specific for transient delivery of preassembled editing machinery and *in vivo* CAR-T generation. Thus, the above research illustrates how the use of receptor-directed delivery and engineered viral envelope enables enhancement of immune cell selectivity and reducing non-specific transduction.

Although viral vectors are known for their efficient delivery, they suffer from different drawbacks, such as pre-existing or induced immunity against viruses, vector-specific cargo limitations, difficulties in repeated administration, complex production processes, and safety issues. Integrating vectors require additional consideration regarding insertional mutagenesis and genome safety. In terms of genome editing applications, long-term expression of nucleases increases the risk of off-target editing compared to transient delivery of preassembled editing machinery (Ge et al., 2024). Consequently, viral and non-viral platforms can complement each other: viral vectors provide efficient and stable genetic manipulation while LNPs and polymeric nanoparticles allow for transient expression, modular design, repeat administration, and avoidance of genomic integration of the vectors.

## 5 *In vivo* engineering of immune cells

With recent breakthroughs in targeting drug delivery technologies, *in vivo* engineering of immune cells has become a rapidly developing therapeutic technology, which allows reprogramming of immune cells in living bodies. Unlike traditional adoptive cell therapy techniques based on the time-consuming procedure of isolation, genetic modification, proliferation, and infusion of modified cells back into the patient’s body, *in vivo* engineering allows for the direct delivery of genetic cargo into innate immune cells (Isaac et al., 2024). Such a technique has a potential to revolutionize manufacturing, making it more cost-effective, faster and accessible without losing efficiency in tumor killing ability. With the development of novel technologies in the field of nanotechnology and synthetic biology, more efficient engineering of T cells, NK cells, macrophages and other immune populations has become possible (Wang et al., 2025). Representative targeted delivery systems for *in vivo* immune cell engineering and their therapeutic applications are summarized in Table 2.

### 5.1 CAR-T cell generation

The emergence of CAR-T cell therapy has revolutionized cancer care with its remarkable capacity to elicit sustained therapeutic responses even in drug-resistant hematologic cancers (Al-Hussaniy et al., 2025). Nevertheless, clinical use of this strategy is severely limited by the challenging nature of *ex vivo* manufacturing which includes several steps such as leukapheresis, viral transduction, massive cell expansion, and quality control tests prior to infusion of the modified T cells. These complicated manufacturing steps result in increased cost of treatment, prolonged time of manufacture and decreased patient access (Li H. et al., 2026). *In vivo* production of CAR-T cells is considered an interesting solution to overcome these problems because it allows delivery of genetic material encoding the CAR directly into the blood stream. Nowadays, different methods including the use of liposomes, antibodies, extracellular vesicles, and peptides can be used to deliver the genetic material for engineering of T cells *in vivo* (Zhao et al., 2026). The choice of mRNA or DNA delivery depends on the specific therapeutic purpose because the first one guarantees temporary expression of the CAR, while the latter is associated with permanent genome modification. Recent preclinical data have shown promising results, suggesting feasibility of *in vivo* CAR-T cell production (Chen, Zhang, et al., 2025b). One of the most promising non-viral methods is NCtx, which was created by Bimbo et al. and represents a novel CD3/CD7-targeting lipid nanoparticle-based delivery system designed to deliver DNA minicircles encoding CARs along with SB100x transposase mRNA. Effective targeting of both resting and activated T cells provided for stable integration of genes, efficient expression of CARs, significant cytotoxic activity against antigens and constant production of cytokines. One intravenous injection led to development of long-lasting CAR-T cells producing durable antitumor effect and extending life expectancy in humanized mice with leukemia. Moreover, NCtx allowed generating dual CD19/CD22 CAR-T cells (Bimbo et al., 2025).

In addition, optimization of transient CAR expression has shown the significance of delivery strategy. Picht et al. examined electroporation versus lipid nanoparticle-based delivery of mRNA for transient CAR-T cell engineering and found that even though electroporation produced high levels of initial CAR expression, it led to the induction of cellular stress response, DNA damage response, and exhaustion of T cells through activation of genes that are responsible for T cell exhaustion. On the other hand, LNP-based delivery maintained the fitness of cells by decreasing the levels of TIM-3 and LAG-3 and retaining their capability to activate, migrate, and kill. Single-cell RNA sequencing analysis showed upregulation of immune effector functions and cytotoxicity in LNP-engineered CAR-T cells (Picht et al., 2026). The use of therapies that reverse the immunosuppressive effect of the tumor microenvironment has contributed to increasing the treatment options of CAR-T therapy in solid tumors. Li et al. developed a bacterial outer membrane vesicle (OMV)-mediated delivery system termed BROAD-CAR, which not only counteracts the immunosuppression of the TME but also tackles the problem of tumor antigen heterogeneity. The use of engineered OMVs that express anti-PD-L1 single chain variable fragment allowed delivering CAR-targeted antigens directly to the tumor cells. The use of this technique has improved the ability of CAR-T cells to identify and kill cancer cells (Li Y.-R. et al., 2026). In sum, these research studies show how developments in the fields of nucleic acid delivery, nanotechnology, and synthetic biology are making it possible to produce CAR-T cells directly in the body.

### 5.2 CAR-NK cell generation

The use of NK cells as an option to T cells appears quite reasonable due to the capability of NK cells to kill cancer cells without priming by antigen and to decrease the probability of graft-versus-host disease in allogeneic conditions. NK cells engineered with chimeric antigen receptors are known to be highly specific to tumor cells and show higher cytotoxicity (Laskowski et al., 2022). Hence, a significant amount of work was done to create a technology of generating CAR-NK cells *in vivo* and to overcome limitations of conventional *ex vivo* engineering of immune cells. Targeted viral delivery is one of the most perspective strategies to achieve this goal (Manzar et al., 2026). Andorko et al. created INT2104, a CD7-targeted lentiviral vector, which enabled selective transduction of endogenous T and NK cells after a single intravenous injection. The engineered immune cells were provided with CARs recognizing CD20 antigens, underwent persistent expansion, completely depleted B-cells, and successfully eliminated CD20-positive tumors in humanized mice without lymphodepletion. Importantly, it was proven in cynomolgus macaques that there is no off-target expression, and this strategy is well tolerated. Thus, this approach enables to generate off-the-shelf *in vivo* CAR-immune cells (Andorko et al., 2025). Enhancement of local activity of CAR-NK can be achieved by combination treatment strategies. Strecker et al. created CAR-NK cells targeted to HER2 protein and combined them with the adeno-associated virus carrying anti-PD-1 immunoadhesin specifically recognizing HER2. This local production of checkpoint inhibitors allowed to counteract the adaptive PD-L1 upregulation in glioblastoma leading to increased antitumor activity, prolonged survival, and complete tumor eradication in certain immunocompetent models (Strecker et al., 2022). However, nonviral methods of engineering were shown to be quite productive as well. Golubovskaya et al. used an mRNA-lipid nanoparticle-based platform which provided extremely high efficiency (up to 78%–95%) of CAR expression in expanded NK cells. Both CD19- and BCMA-specific CAR-NK cells showed higher cytotoxicity towards leukemia and multiple myeloma respectively as well as higher production of IFN-γ and granzyme B. *In vivo*, CD19 CAR-NK cells resulted in inhibition of leukemia progression thus showing the potential of CAR-NK generation using mRNA-LNP platform (Golubovskaya et al., 2023). There are also quite promising methods related to genetic optimization of immune cell fitness. Shaik et al. created GPC3-specific CAR-NKT cells overexpressing transcription factor BATF3 which leads to higher metabolic fitness, proliferation, persistence and enrichment of CD62L-positive NKT cells. Overexpression of BATF3 improved functionality and provided long-lasting tumor control and prolonged survival in hepatocellular carcinoma models (Shaik et al., 2022). In addition, there are complementary strategies which aim at improvement of CAR immune cells’ persistence and activity. Grzywa et al. developed an amphiphilic peptide mimotope vaccine enabling selective restimulation of clinically relevant CAR-T cells *in vivo*. Vaccine-induced stimulation led to persistent expansion of CAR-T cells, higher production of cytokines, improvement of CAR-T-cell persistence and prolonged survival in leukemia and lymphoma models. Thus, this strategy provides a broad method of enhancing CAR-cell activity after their adoptive transfer (Grzywa et al., 2026). Simultaneously, Koam et al. created cholesterol-modified amphiphilic polyaspartamide nanoparticles which allowed efficient delivery of mRNA into NK cells. The designed polymer improved intracellular delivery of mRNA, increased expression of NKG2D, increased IFN-γ production and increased NK cells’ cytotoxicity towards colon cancer cells without toxicity. These data provide evidence of the possibility of polymeric nanoparticles to act as carriers for NK cells engineering (Koam et al., 2025). Together, these studies demonstrate that targeted viral vectors, LNPs, polymeric carriers, and biomaterial-based engineering approaches are rapidly expanding the feasibility of *in vivo* CAR-NK generation while improving immune-cell persistence, metabolic fitness, and therapeutic efficacy.

### 5.3 CRISPR and mRNA-Based immune reprogramming

In addition to CAR engineering, delivery technologies have been applied to modify the function of immune cells using the approach of mRNA therapy and CRISPR-based genome editing. mRNA allows the delivery of cytokines, transcription factors, receptors, and signaling proteins without any modification of the genome, and thus provides good safety for the treatment of cancers (Dong et al., 2026). On the other hand, CRISPR technology makes it possible to precisely edit the regulation of immunity, which includes the disruption of inhibitory immune checkpoints as well as introduction of therapeutic genes that improve the survival and functionality of immune cells. In recent research, it has been shown that the targeted delivery of CRISPR ribonucleoprotein and mRNA allows for reprogramming of endogenous immune cells *in vivo* without difficulties related to *ex vivo* cell manufacturing (Lei et al., 2024). However, clinical application of such strategies requires the further improvements in delivery specificity, intracellular targeting and trafficking, editing specificity, and long-term safety. The example of immune cell reprogramming, which does not include classical genome editing, has been provided by Ma et al. These scientists have shown that the expression of CD5-targeting CAR leads to the rapid internalization and degradation of CD5 on engineered T-cells and prevents fratricide because there is no need to knockout CD5 gene. Although the temporary reduction of CD5 expression occurs on both malignant and normal T cells due to the interaction between CAR and its antigen, the tumor cells remain sensitive to killing. According to clinical studies, there was a significant regression of tumors and preservation of healthy CD5-negative T cells, which means that CAR-induced antigen modulation is a valuable way to improve the activity of CAR-T cells without any permanent changes in genome (Ma et al., 2024). Overall, the integration of mRNA therapeutics, precision genome editing, and advanced delivery technologies is rapidly expanding the capabilities of *in vivo* immune cell engineering. Continued innovation in targeted delivery platforms and programmable genetic systems is expected to accelerate the development of safer, more effective, and broadly accessible next-generation cancer immunotherapies.

## 6 Applications in cancer immunotherapy

*In vivo* immune cell engineering is emerging as a promising strategy for cancer immunotherapy by reducing the need for complex *ex vivo* manufacturing. Targeted delivery systems can introduce mRNA, DNA, gene-editing machinery, or immunomodulators directly into endogenous immune cells, enabling repeated dosing and transient or durable immune reprogramming (Ren et al., 2024). Although most approaches remain preclinical, encouraging antitumor activity has been demonstrated in hematological and solid tumors, including through combination with checkpoint blockade and tumor microenvironment modulation. Beyond T cells, targeted platforms are being developed to reprogram myeloid cells (Rosa et al., 2026). Nie et al. developed mRNA-LNP-Ery, an erythrocyte-based system that preferentially delivers mRNA to splenic CD11b^+^ myeloid cells while reducing liver accumulation. The platform generated HER2- or CD19-specific CAR myeloid cells *in vivo*, enhanced CD8^+^ T-cell and NK-cell tumor infiltration, and produced potent antitumor effects at lower mRNA doses than conventional LNPs, with a favorable safety profile (Nie et al., 2026). Precision *in vivo* genome engineering is also advancing rapidly. Nyberg et al. combined Cas9 ribonucleoproteins with T-cell-targeted AAV donor vectors for site-specific CAR integration into the TRAC locus. The anti-CD3-EDV/AAV-hT7 system generated up to ∼20% CAR-T cells *in vivo*, which showed robust expansion and memory-like properties, resulting in B-cell aplasia, durable tumor regression, and resistance to rechallenge in a B-cell acute lymphoblastic leukemia model (Nyberg et al., 2026).

### 6.1 Hematologic malignancies

Hematologic malignancies are particularly suitable for *in vivo* immune-cell engineering because tumor cells are accessible in circulation and clinically validated antigens such as CD19, CD22, and BCMA are available. Although conventional CAR-T therapies have transformed the treatment of B-cell leukemias, lymphomas, and multiple myeloma, their high cost, complex manufacturing, and patient-to-patient variability remain major limitations. Targeted delivery systems offer an alternative by generating CAR-T cells directly *in vivo* with the potential for repeated dosing (Zhang et al., 2022). Lemgart et al. developed CD8-targeted LNPs that selectively deliver CD22 CAR mRNA to CD8^+^ T cells, generating >20% CAR^+^ CD8^+^ T cells *in vivo* with minimal off-target transfection. The engineered cells showed potent cytotoxicity and nearly complete B-cell depletion, while repeated dosing reduced Nalm6 leukemia burden without significant toxicity or cytokine-related adverse events (Lemgart et al., 2026). Similarly, Sun et al. developed GT801, a tLNP platform delivering anti-CD19 CAR mRNA to T cells using CLAMP antibody conjugation. GT801 maintained CAR expression for >14 days with <3% transfection of monocytes, macrophages, or dendritic cells. A single low-dose intravenous administration produced >95% B-cell depletion and >30-fold CAR-T expansion with low cytokine release, supporting its potential as a scalable, re-dosable platform (Sun et al., 2025). Genome editing can further enhance CAR-T function and persistence. Using CRISPR loss-of-function screening, Knudsen et al. identified CDKN1B as a negative regulator of BCMA CAR-T cells. CDKN1B knockout enhanced proliferation, effector function, tumor infiltration, persistence, and survival while reducing exhaustion under chronic antigen stimulation (Knudsen et al., 2025). Myeloid cells can also be engineered *in vivo*. Nie et al. used mRNA-LNP-Ery to generate HER2- or CD19-specific CAR myeloid cells through targeted delivery to splenic CD11b^+^ cells. These cells promoted T- and NK-cell infiltration and tumor suppression at one-tenth the conventional LNP dose with reduced systemic toxicity (Nie et al., 2026).

### 6.2 Solid tumors

Solid tumors present major barriers to immune-cell therapy, including poor immune-cell trafficking, dense stroma, antigen heterogeneity, hypoxia, and an immunosuppressive TME. Targeted delivery platforms may overcome these limitations by reprogramming immune cells *in vivo*, enhancing tumor infiltration and cytotoxicity, and simultaneously modulating the TME (Torabi et al., 2025). Several targeted LNP (tLNP) platforms have demonstrated *in vivo* CAR-T generation. Hunter et al. developed CD8-targeted L829-tLNPs that deliver CAR mRNA directly to CD8^+^ T cells, inducing B-cell depletion and antitumor activity in humanized models and sustained B-cell depletion followed by naïve B-cell repopulation in cynomolgus monkeys (Hunter et al., 2025). Moreover, Bimbo et al. developed NCtx, a dual CD3/CD7-targeted platform delivering minicircle DNA and transposase mRNA to generate durable CAR-T cells, resulting in tumor regression and improved survival in humanized leukemia models (Bimbo et al., 2025). In turn, Chen et al. developed an Fc-binding nanobody-based antibody-capture LNP system that improved antibody orientation and achieved >1,000-fold higher protein expression with selective T-cell mRNA delivery (Chen M. Z. et al., 2025). Similarly, antibody-functionalized extrahepatic LNPs delivered CD19 CAR mRNA to T cells and generated transient, dose-dependent CAR-T cells capable of depleting up to 90% of circulating B cells (Billingsley et al., 2024). Notably, Kim et al. identified CD4^+^ T cells as major targets of LNP-mRNA vaccines in lymphoid tissues, where they efficiently internalized LNPs, expressed encoded proteins for several weeks, and supported antigen-specific antibody and neutralizing responses (Kim et al., 2025). Engineered NK and innate-like lymphocytes provide additional strategies for solid tumors. Li et al. developed IL-15-enhanced mesothelin-targeted allogeneic CAR-NKT cells that combine CAR-dependent recognition with NK receptor-mediated killing. These cells improved tumor infiltration, reduced exhaustion, and prolonged survival in pancreatic cancer models while showing low immunogenicity and minimal toxicity (Li et al., 2025). Wang et al. developed DNA nanochannel-engineered artificial receptors (NCARs) that enhanced NK-cell infiltration, retention, and antigen-independent cytotoxicity, suppressing melanoma growth despite antigen heterogeneity and immunosuppression (Wang D. et al., 2026). Transient NKG2D-DAP12 CAR mRNA-engineered NK cells also demonstrated enhanced activity against metastatic colorectal cancer, with a pilot clinical study reporting reduced malignant ascites and tumor burden and a complete metabolic response in one treated liver lesion (Xiao et al., 2019). Localized cytokine delivery can further enhance antitumor immunity while limiting systemic toxicity. Jiang et al. developed U-101 LNPs carrying tumor-activated IL-2 mRNA that remains inactive systemically and is activated by MMP-14 within tumors. In MC38 tumors, treatment achieved 70.3% tumor growth inhibition, increased intratumoral IL-2, IFN-γ, and CD8^+^ T-cell infiltration, limited Treg expansion, and reduced systemic toxicity compared with conventional IL-2 therapy (Jiang et al., 2024).

### 6.3 Combination with immune checkpoint blockade

Combining intratumoral immune-cell engineering with immune checkpoint blockade is a promising strategy to overcome tumor immunosuppression and improve therapeutic durability. Although engineered immune cells can induce potent antigen-specific cytotoxicity, their activity is often limited by inhibitory pathways such as PD-1/PD-L1, CTLA-4, and CD47/SIRPα. Targeted delivery platforms can address these barriers through localized checkpoint inhibition, co-delivery of immunostimulatory agents, or genetic modification of immune cells (Rossetti et al., 2022). Liu et al. developed hybrid cell-membrane nanovesicles displaying PD-1 and SIRPα receptors to simultaneously block PD-1/PD-L1 and CD47/SIRPα signaling. This nanovesicles enhanced macrophage phagocytosis, CD8^+^ T-cell infiltration, dendritic-cell maturation, and M1 macrophage polarization. In B16F10 melanoma, treatment suppressed tumor growth by 77%, increased TNF-α and IFN-γ, and showed no significant organ toxicity (Liu et al., 2024). Similarly, Mujahid et al. conjugated anti-PD-1 antibodies to PEGylated mesoporous polydopamine nanoparticles, improving intratumoral retention and checkpoint blockade. In CT26 colorectal tumors, intratumoral treatment reduced tumor growth, achieved complete regression in 37.5% of mice, prolonged survival, and enhanced infiltration and activation of CD8^+^ T cells, NK cells, dendritic cells, and M1 macrophages (Mujahid et al., 2025). Overall, these studies demonstrate the potential of *in vivo* immune-cell engineering for hematologic and solid tumors, particularly in combination therapies. Further improvements in cell targeting, intracellular delivery, immune activation, and safety will be essential for clinical translation.

## 7 Clinical translation and future perspectives

However, with the rapid progress of targeted delivery technologies, *in vivo* immune cell engineering moves from proof-of-concept experiments to the clinic. While existing adoptive cell therapies provide sufficient therapeutic outcomes, the ability to edit the immune cells right inside the body holds promises to make cell therapy manufacturing more efficient, cheaper, and more accessible (Gao B. et al., 2026). Still, there are numerous scientific and safety considerations which should be resolved before implementing targeted *in vivo* delivery technologies in clinical routine.

### 7.1 Current clinical studies

While most targeted *in vivo* immune cell engineering methods are still in preclinical studies, the progress of the field is such that its technologies become closer to being tested in clinical trials. The success of non-viral nucleic acid delivery platforms based on liposomes, such as mRNA in COVID-19 vaccines, proves the safety, scalability, and clinical viability of those approaches, providing good background for using the same technology in cancer immunotherapy. Further progress made by preclinical research shows that antibody-targeted LNPs can specifically deliver mRNA, DNA, siRNA, or gene-editing cargo to the immune cells, thus reprogramming them *in vivo* without *ex vivo* manufacturing (Billingsley et al., 2024).

Recently, the field entered an important phase of clinical translation. Particularly, the targeted non-viral *in vivo* platform for BCMA-specific CAR-T generation–ESO-T01 – has become the first of its kind to enter early clinical evaluation in relapsed or refractory multiple myeloma (Gao Y. et al., 2026). This achievement marks an important point for translating targeted immune-cell engineering into clinical practice and provides the first chance to test the safety and therapeutic value of *in vivo* CAR-T production in humans. Besides the CAR-T therapies, several clinical-stage RNA therapeutic platforms utilizing nanoparticles target *in vivo* modulation of tumor immune microenvironment, delivering mRNA, siRNA, immune agonists, or gene editing components to enhance antigen presentation, reverse immune suppression, T-cell activation, and increase the effectiveness of immune checkpoint inhibitors. Despite most of those technologies are still in early clinical development, promising preclinical efficacy and improved clinical safety profiles show that targeted non-viral delivery platforms are starting to become a viable alternative to traditional viral vector- or *ex vivo* manufacturing-based cell therapies.

### 7.2 Safety and regulatory challenges

Despite the promising therapeutic potential, several biological, technical, and regulatory hurdles must be overcome before wide-scale clinical adoption of *in vivo* immune cell engineering platforms can take place. First, off-target delivery, which may decrease the effectiveness of the treatment and increase the chances of adverse biological effects, remains one of the biggest hurdles associated with this technology. However, thanks to recent advances in antibody-conjugated LNPs and receptor-targeted delivery systems, the degree of immune cell specificity has greatly increased (Billingsley et al., 2024). Immune toxicity is another important safety factor that should be considered. The formulation of nanoparticles containing mRNA, siRNA, or gene-editing cargoes may activate innate immunity, trigger cytokine production, complement cascade, and cause anti-drug immune responses upon repeated administration. For this reason, nanoparticle composition, surface properties, dosage, and target-specific ligands should be carefully optimized to maximize the effectiveness of treatment and minimize the risks of triggering a systemic inflammatory response (Hamilton et al., 2023). Safety is especially important for genome-editing based therapies as long-term genomic safety should be assessed prior to the introduction of these technologies to the clinic. Although recent delivery platforms have been highly efficient and virtually free of acute toxicity, the comprehensive assessment of off-target genome editing, chromosomal rearrangements, large deletions, and persistence of engineered immune cells is still necessary. Recent peptide-assisted genome editing and targeted delivery platforms have shown great effectiveness with no significant transcriptional changes (Zhang et al., 2024). Regulatory aspects are also challenging because of the combined nature of *in vivo* immune engineering products that include cell and gene therapy as well as advanced nanomedicine. Regulators are likely to demand comprehensive assessment of biodistribution, pharmacokinetics, manufacturing reproducibility, genome-editing accuracy, persistence of transgene expression, immunogenicity, and long-term safety of these therapies before any approval is granted. With the further development of targeted and non-viral delivery systems, standardization of manufacturing procedures, potency assays, and long-term monitoring is required (Billingsley et al., 2024; Bimbo et al., 2025).

Apart from biological factors, manufacturing and scaling issues remain key barriers to clinical translation. Even though *in vivo* engineering may obviate the need for patient-specific *ex vivo* cell manufacturing, targeted delivery platforms require consistent large-scale manufacturing, including nanoparticle synthesis, ligand conjugation, drug loading, stability, and quality control under GMP conditions. This problem may be especially challenging for multifunctional systems that include targeting antibodies, nucleic acids, and special lipids or polymers. Thus, the potential cost savings of *in vivo* engineering will ultimately depend on the ability to manufacture these systems reliably and economically at a clinical scale. One another important barrier to clinical translation is to translate preclinical results to humans. The vast majority of *in vivo* immune engineering approaches have been studied in mice and humanized mouse models, which do not reflect human receptors, immune cells distribution, nanoparticles distribution, innate immune response, and pharmacokinetics. Differently populated species, varying in immune cell populations, receptor numbers, body sizes, and pre-existing immunity, may considerably impact the targeting efficacy, therapeutic efficacy, and safety of the approaches. Thus, preclinical validation in relevant large animal models and thorough evaluation of biodistribution, dose response, pharmacokinetics, immunogenicity, and repeated dosing toxicity are crucial for successful clinical translation.

### 7.3 Future directions

Further development of *in vivo* immune cell engineering will depend on improvements in the area of targeted drug delivery, synthetic biology, and genome engineering. Modern advancements in the field of LNPs, biomimetic nanocarriers, antibody-based delivery systems, and CPPs made the delivery of mRNA, siRNA, proteins, and genome-editing cargos to the immune cells very precise and efficient (Zhu et al., 2024). Thus, these innovative delivery platforms allow direct *in vivo* cell engineering with the potential to reduce treatment-related adverse effects and may become a valuable alternative to classical *ex vivo* production of genetically modified immune cells. At the same time, rapid advancements in the field of mRNA technology, CRISPR-based genome engineering, base and prime editing, and programmable gene circuits will increase the capabilities of the genetically engineered immune cells. With the help of precise delivery system and programmable genetic modification tools, it will be possible to achieve dynamic regulation of immune-cell activation, persistence, homing, and tumor recognition, thereby increasing their therapeutic potency while reducing treatment-related adverse effects (Bimbo et al., 2025; Hunter et al., 2025). Another trend will be creation of multivalent delivery platforms that can integrate multiple modalities into one nanocarrier. It will make it possible to deliver the cargos that encode CARs, gene-editing machinery, cytokines, immune agonists, small-molecules drugs, and other components to the tumor tissue and remodel the immune suppressive tumor microenvironment to increase antitumor activity. Multivalent combinatorial delivery platforms are already proven in preclinical studies and promise to provide more effective clinical outcomes compared to the monomodal approaches (Hamilton et al., 2023; Li et al., 2025). Furthermore, another tool that may dramatically speed up the development of next-generation delivery systems is artificial intelligence (AI) and machine learning (ML). Computational approaches are increasingly used for optimization of ionizable lipids, polymer molecules, targeting ligands, and nanoparticle structures via prediction of their properties. This will help to significantly decrease the development process and to improve the clinical translation of the targeted delivery platforms. Overall, all these advancements make *in vivo* immune cell engineering one of the most promising next-generation techniques in cancer immunotherapy. By combining precision delivery technologies, programmable genetic engineering, and synthetic immunology, the rapidly developing field can overcome some of the limitations of the current generation of adoptive cell therapies, such as complexity of the production, high cost, and low accessibility of the treatment. Interdisciplinary collaboration between biomaterial scientists, immunologists, synthetic biologists, clinicians, and regulatory bodies will be crucial for full implementation of these technologies.

There are still many important questions that need to be answered. It is not yet known which receptors on the immune cells offer the best combination of specificity and efficiency of uptake, how the efficiency of delivery seen in rodents can be extrapolated to humans, or how much and for how long the *in vivo* engineering needs to take place to have a beneficial effect. Also, the pros and cons of transient versus permanent reprogramming need to be assessed in the light of efficacy, repeated dosing, immunogenicity, and genomic safety in the long term.

## 8 Conclusion

The field of *in vivo* engineering of immune cells has been emerging very fast, and there are indications that the technology could potentially transform the future of cancer immunotherapy. This is because the technology could provide a more accessible method compared to *ex vivo* manufacturing of adoptive cellular therapies by offering a simpler and less expensive way of manufacturing therapeutic immune cells directly in the body. Recently, significant advances in cell-penetrating peptides, antibody and receptor targeting mechanisms, liposomes, polymers, biomimetic materials, and LNPs have facilitated more effective and selective delivery of mRNA, gene editing components, proteins, and other therapeutic cargoes to immune cells. These developments have contributed greatly to the ability to engineer various immune cells such as T-cells, NK cells, dendritic cells, and macrophages *in vivo* and applications that include immune modulation to expression of CAR and gene editing. Among the available delivery technologies, LNPs have proven to be one of the most clinically advanced non-viral delivery platforms for nucleic acid due to the success of nucleic acid drugs and their ability to deliver the cargo to immune cells selectively. At the same time, antibody-mediated, receptor-mediated, and biomimetic delivery systems have enhanced targeting and specificity to immune cells, as well as increased cellular internalization and decreased systemic toxicity. Thus, the convergence of delivery technologies with recent advances in mRNA therapeutics, genome editing based on CRISPR, synthetic biology, and programmable gene circuits is making it possible to exert greater control over the functions of immune cells.

Notwithstanding the advances made, several challenges remain to be addressed before wide-scale use in clinic becomes feasible. These challenges include improvement of target specificity, overcoming biological barriers like endosome entrapment, reduction of off-target delivery, immune-related toxicities, ensuring long-term genotoxicity safety, and setting up manufacturing process. Meeting all these challenges requires a collaborative effort between different experts including those working in the fields of biomaterials, immunology, synthetic biology, clinical practice, and regulation. Going forward, the convergence of nanotechnology, precision medicine, synthetic immunology, artificial intelligence-driven materials design, and genome engineering is expected to further fuel this development. When these technologies mature and become clinically ready, they will help overcome some of the limitations of existing cell-based therapies and create a new generation of off-the-shelf, programmable, and personalized immune-based treatment modalities. Therefore, targeted *in vivo* immune cell engineering might emerge as a broadly applicable therapeutic platform that will improve treatment options for patients with hematologic cancers and solid tumors while increasing global accessibility of next-generation cancer immunotherapies.
